# Loss of protein phosphatase 1 regulator TIMAP protein triggers EMT in A549 cells

**DOI:** 10.1186/s12964-026-02873-5

**Published:** 2026-04-13

**Authors:** Fanni Szalmás, Lilla Nagy, Peter Bai, Anita Boratkó

**Affiliations:** 1 Department of Medical Chemistry, Faculty of Medicine, University of Debrecen, Egyetem tér 1, Debrecen, H-4032 Hungary; 2 MTA-DE Lendület Laboratory of Cellular Metabolism, Debrecen, Hungary; 3 HUN-REN Cell Biology and Signaling Research Group, Debrecen, 4032 Hungary; 4 Research Center for Molecular Medicine, Faculty of Medicine, University of Debrecen, Debrecen, 4032 Hungary

**Keywords:** EMT, TIMAP, A549 lung adenocarcinoma, Invasiveness, Cell signalling, Cancer stem cell phenotype

## Abstract

**Background:**

Epithelial–mesenchymal transition (EMT) is a cellular reprogramming process that enables epithelial cells to acquire mesenchymal traits, leading to enhanced motility, invasiveness, and resistance to therapy. Transforming growth factor-β-inhibited, membrane-associated protein (TIMAP) is a regulatory subunit of protein phosphatase 1. Altered TIMAP expression has been implicated in tumor progression, suggesting a potential role in EMT.

**Methods:**

Human A549 lung adenocarcinoma cells were subjected to stable lentiviral shRNA-mediated knockdown of TIMAP. Phenotypic, molecular, and functional consequences of TIMAP depletion were assessed using Western blotting, RT-qPCR, high-content imaging, electric cell–substrate impedance sensing, RNA sequencing with pathway enrichment, cytokine array profiling, 3D spheroid assays, trans-endothelial migration assays and metabolic activity measurements. EMT-associated signalling pathways, including TGF-β/SMAD, Wnt/β-catenin, and PI3K/Akt, were examined. Expression and survival correlations were evaluated using publicly available lung adenocarcinoma datasets.

**Results:**

TIMAP knockdown was confirmed at both transcript and protein levels and induced pronounced morphological changes toward a mesenchymal-like phenotype. shTIMAP cells exhibited enhanced attachment, accelerated wound closure, and increased transendothelial migration. Transcriptomic profiling revealed enrichment of EMT-associated pathways. shTIMAP cells displayed reduced epithelial protein markers such as E-cadherin and ZO-1 and increased mesenchymal markers like N-cadherin, Snail, and Slug expression. Activation of EMT-related signalling, including elevated phosphorylation of SMAD2/3, β-catenin, and Akt was also observed. Re-expression of TIMAP or pharmacological inhibition of the TGF-β pathway reversed EMT phenotype. TIMAP depletion altered the secretory profile toward pro-inflammatory, pro-invasive cytokines and disrupted 3D spheroid architecture, promoting spheroid disintegration and invasive outgrowth. TIMAP depletion shifted cellular metabolism from oxidative phosphorylation towards glycolysis and induced cancer stem cell phenotype, with upregulation of CD44, CD133 and downregulation of CD24. Clinically, TIMAP expression is significantly downregulated in lung adenocarcinoma already in early-stage disease, and low tumoral TIMAP levels predict poor survival.

**Conclusion:**

Loss of TIMAP promotes EMT, enhances motility and invasion, and induces pro-tumorigenic signalling and secretory changes in A549 lung adenocarcinoma cells. These effects likely contribute to the association between low TIMAP expression and adverse patient outcomes, highlighting TIMAP as a potential prognostic biomarker and therapeutic target in lung cancer.

**Graphical abstract:**

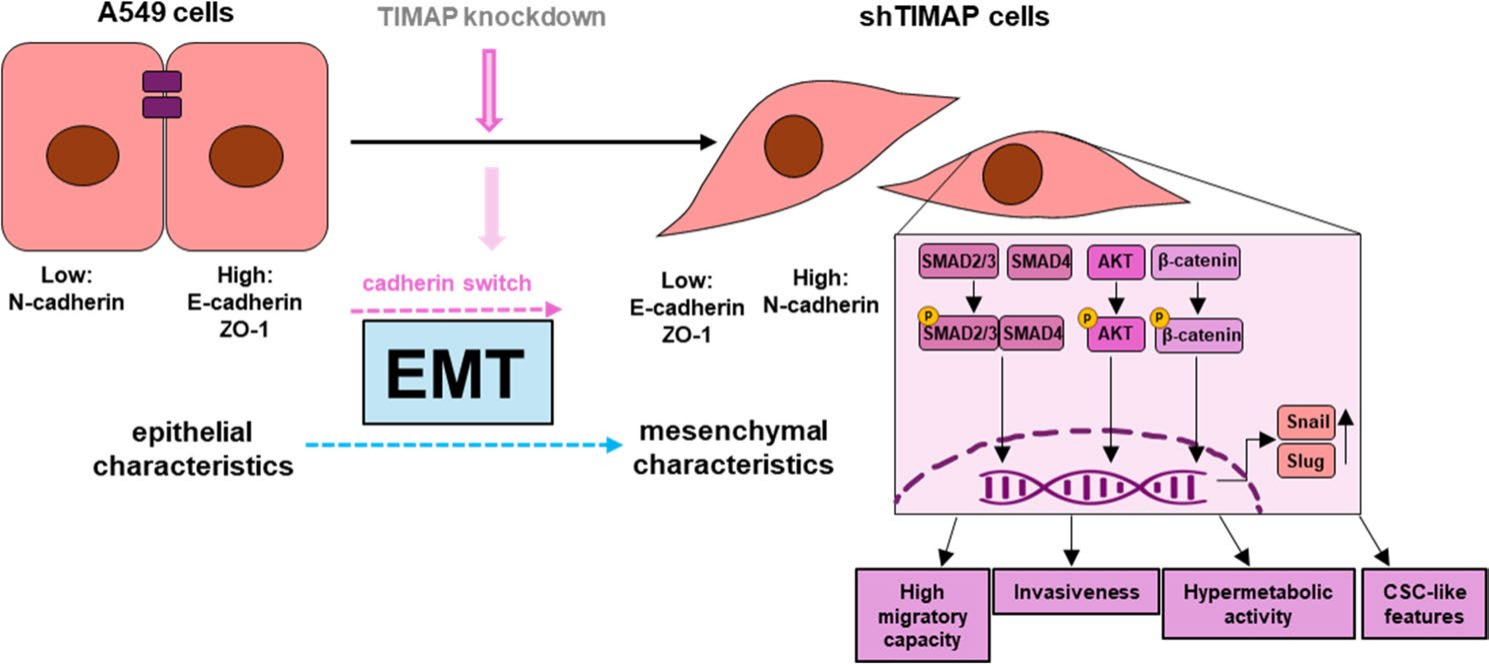

**Supplementary Information:**

The online version contains supplementary material available at 10.1186/s12964-026-02873-5.

## Background

Epithelial-mesenchymal transition (EMT) is a well-recognized cellular program in which epithelial cells lose their polarity and adhesion and gradually acquire mesenchymal traits [1]. This concept was first introduced by Greenburg and Hay, describing how epithelial cells elongate and lose their cell-cell junctions [2]. During EMT, epithelial cells undergo a complex reprogramming process in which they lose their epithelial characteristics and acquire a mesenchymal phenotype. Epithelial cells are characterized by cell-cell interactions, in the form of tight junctions, adherent junctions, and desmosomes and have a defined apical-basal polarity. In contrast, mesenchymal cells are more loosely attached, lack polarity, and surrounded by the extracellular matrix (ECM) [3, 4]. EMT can be categorized into three types based on the context in which it occurs. The first type happens during embryonic development where it is essential for tissue and organ formation. The second type is involved in tissue regeneration and wound healing. The third type can be observed in pathological conditions such as tumor and metastasis formation. Unlike the first two types, EMT in tumorigenesis is often dysregulated, that contributes to poor prognosis in solid tumors that metastasize more easily and have higher invasiveness [5, 6]. In cancer, primary tumors are characterized by high rates of cell proliferation and angiogenesis. As tumors progress, cells undergoing EMT gain invasiveness, enabling them to break the basement membrane followed by the lymphohematogenous dissemination. The role of EMT in tumor metastasis has been shown in numerous tumor types [1, 7], including lung adenocarcinoma, where EMT led to the formation of malignant phenotypes and poor clinical outcomes [6, 8–10]. Since EMT is a reversible process, the deeper level understanding and regulation of EMT offer valuable insight for developing therapeutic strategies to prevent tumor metastasis [11].

During EMT, molecular changes occur both at the gene expression and protein levels. EMT is primarily driven by so called EMT-activating transcription factors (EMT-TFs), including members of the Snail, TWIST and ZEB families. These TFs contribute to the loss of epithelial characteristics through the downregulation of epithelial markers like occludins, claudin and E-cadherin and the disruption of adherens junctions. Simultaneously, mesenchymal characters are enhanced, marked by increased expression of N-cadherin, vimentin and matrix metalloproteinases (MMP-2, -3, -9) [12]. The transcriptional repression of E-cadherin by factors like Snail results in the release of β-catenin from the membrane into the cytoplasm [13]. This free β-catenin then translocate into the nucleus and associates with lymphoid enhancer factor/T cell factor (LEF/TCF) TF [4, 14].

EMT is not defined by a single pathway, but is instead induced by various pathways, including the transforming growth factor (TGF)-β, bone morphogenetic protein (BMP), Wnt/β-catenin, NOTCH, Shh, and receptor tyrosine kinases [14–16]. Among these, the TGF-β pathway is one of the most extensively studied [17]. It induces EMT through the activation of Smad proteins, and enhanced expression of Smad2 or Smad3 with Smad4 has been shown to promote EMT [18]. Canonically, TGF-β ligands bind to their receptors (TGFBR1/2), leading to phosphorylation of receptor-regulated Smads (Smad2/3), which then form complexes with Smad4 and translocate to the nucleus to regulate EMT-related gene expression, including SNAI1, SNAI2, ZEB1, and ZEB2, triggering profound changes in gene expression, cytoskeletal organization, and cell–cell adhesion [19, 20]. In parallel, TGF-β activates non-canonical pathways such as phosphoinositide 3-kinase (PI3K)/Akt, mitogen-activated protein kinase (MAPK), and Rho/ROCK, amplifying its effects on motility, invasion, and extracellular matrix remodelling [21]. The gene PPP1R16B is downregulated in response to TGF-β1 treatment, hence the corresponding protein was named TGF-β-inhibited membrane-associated protein, TIMAP [22]. TIMAP belongs to the myosin phosphatase targeting protein (MYPT) family, which are regulatory subunits of protein phosphatase 1 (PP1). PP1 holoenzymes consist of a catalytic subunit (PP1c) that binds to regulatory proteins such as TIMAP, directing the holoenzyme to specific cellular locations and substrates. The large number of regulatory subunits compared to a relative few phosphatase catalytic subunits underline the central role of the regulatory proteins in determining substrate specificity and subcellular localization [23, 24]. Structurally, TIMAP contains a putative bipartite nuclear localization signal supporting its presence in the nucleus as well [25]. Its intrinsically disordered C-terminal region carries a CAAX-box motif, which undergoes farnesylation, anchoring TIMAP to plasma membrane [26]. This dual targeting enables TIMAP to function in both the nuclear and membrane compartments of the cell. TIMAP is subjected to phosphorylation by protein kinase A (PKA), glycogen synthase kinase 3β (GSK3β) and protein kinase C (PKC), and its phosphorylation status influences both the substrate specificity and activity of the TIMAP-PP1c complex, as well as the localization of the holoenzyme [27, 28]. The interactome and substrate pool of TIMAP e.g. non-integrin laminin receptor 1 (LAMR1) [29, 30], endothelin converting enzyme 1 (ECE-1) [31], ezrin-radixin-moesin (ERM) [22], merlin [32] is associated with physiological processes as cell barrier function, migration and angiogenesis [28]. However, the same proteins are associated with neoplasia-linked processes, as cell proliferation, contact inhibition, cell-cell adhesion or suppressibility by TGF-β1 [28, 33, 34]. In this study we set out to investigate the significance of TIMAP in LUAD pathology using A549 cell model.

## Methods

### Cell cultures

A549 (human Caucasian lung adenocarcinoma epithelial cell line, ATCC CCL-185), SK-LU-1 (human lung adenocarcinoma epithelial cell line, ATCC HTB-57) and tsA201 (a transformed HEK293 cell line) (ECACC, Cat# 96121229) cells were cultured in low-glucose Dulbecco’s Modified Eagle’s Medium (DMEM, 1 g/L glucose) supplemented with 10% heat inactivated foetal bovine serum (FBS) and 2 mM L-glutamine. Stable A549 and SK-LU-1 cells with TIMAP knockdown (shTIMAP) and vector control cells transduced pLKO.1 cells were cultured under the same conditions, with the addition of 1 µg/mL puromycin for selection. Bovine pulmonary artery endothelial cells (BPAEC) were cultured as described earlier [26]. All cells were incubated at 37 °C in a humidified incubator with CO_2_.

### Gene silencing

Lentiviral mediated gene knockdown was utilized to generate a stable TIMAP silenced A549 and SK-LU-1 cell lines. Five TIMAP-specific shRNAs, designed by The RNAi Consortium (TRC; Dharmacon, Catalog ID: RHS4533-EG2605), were used individually. The target sequences were as follows: TRCN0000006916 (sh16; ATAGCAAGAGTTAAATACAGC; 3′UTR), TRCN0000006917 (sh17; TTAGGGTCCTTATTCTCTTGG; ORF), TRCN0000006918 (sh18; TACTGTGCCCATTTCTTCAGC; ORF, 5′UTR), TRCN0000006919 (sh19; TGACCGTGTAATATACCGAGG; ORF), and TRCN0000006920 (sh20; AAAGTTGTCGATGCAGCACTG; ORF, 5′UTR). For viral transduction, tsA201 cells were co-transfected with pLKO.1 plasmids encoding each of the five TIMAP-targeting shRNA sequences (500 ng/µl) together with the packaging and envelope plasmids HDM-Hgpm2, RC-CMV/Rev, and HDM-tat1b (25 ng/µl each), along with HDM-VSV-G (50 ng/µl), using Lipofectamine 3000 transfection reagent (Invitrogen, L3000015). A pLKO.1 Empty Vector Control (500 ng/µl) was used as a negative control (Cat: RHS4080). Viral supernatants were collected 96 h post-transfection, filtered through 0.45-µm pore size membranes, and subsequently utilized for transduction. A549 or SK-LU-1 cells were seeded into 24 well plates at approximately 60% confluence in complete DMEM. The following day, A549 or SK-LU-1 cells were incubated with the virus-containing supernatant supplemented with 8 µg/ml hexadimethrine bromide (polybrene). After a 48-hour incubation, transduced cells were selected in complete medium using containing puromycin (1 µg/ml) for two weeks, replenishing the media every two days. Silencing efficiency was confirmed by qPCR and Western blot analysis.

### High-content analysis

pLKO.1 and shTIMAP A549 cells were seeded into CellCarrier Ultra 96-well microplates for imaging analysis. Cells were stained with DAPI (nuclei), β-tubulin or β-catenin (Alexa 488) and Texas Red phalloidin (F-actin). Imaging was performed with the Opera Phenix high-content screening system (Perkin Elmer, Waltham, MA, USA) utilizing a 63× objective. For evaluation of cell morphology, nuclei were first identified based on DAPI staining, and the cytoplasm based on actin staining. Edge objects were excluded from the analysis to avoid partial cells. Acquired images were quantitatively analysed using Harmony software (version 4.8, Perkin Elmer) integrated with the imaging system [35]. For β-catenin subcellular localization cell were stained with β-catenin specific antibody. Nuclei were identified using the *Find nuclei* algorithm, based on DAPI staining. Nuclear β-catenin intensity was quantified within the defined nuclear mask. Total cellular β-catenin intensity was measured per cell, and nuclear accumulation was expressed as the ratio of nuclear to total β-catenin staining intensity x 100.

### Magnetic 3D cell culturing

3D cell cultures were made by using the 96-well Bioprinting Kit from Greiner Bio-One (Kremsmünster, Austria), in accordance with the protocol provided by the manufacturer. Spheroid formation was performed as previously described [36]. Briefly, NanoShuttle (1 µl/10,000 cells) was added to pLKO.1 control and shTIMAP A549 cells. After incubation, magnetized cells were seeded into cell-repellent surface plates (Cat: 655970; Greiner Bio-One, Kremsmünster, Austria) and placed on a magnetic plate for 3 h to promote spheroid formation. Once the spheroids had formed, the magnetic plate was removed, and images were captured immediately (0 h) and at various time points with a Leica microscope equipped with a Leica MC120 HD camera. Spheroid size and cell migration from the spheroid core were quantified using ImageJ software as described in [36].

### MTT assay

MTT (3-[4,5-dimethylthiazolyl-2]-2,5-diphenyltetrazolium bromide) assay were performed to assess cell viability, as the reduction of MTT to formazan crystals by mitochondrial dehydrogenases correlates with the number of metabolically active, viable cells. Cells were seeded in a flat-bottomed 96-well microtiter plates and allowed to adhere. At the indicated time points, MTT reagent was added to each well at a final concentration 0.5 mg/ml and incubated at 37 °C in a humidified atmosphere for 1 h. The medium was aspirated, and the resulting formazan crystals were solubilized in DMSO. After a 5 s shaking step, absorbance was measured at 540 nm with Thermo Scientific Multiskan GO plate reader.

### Plasmid transfection

pLKO.1 control and shTIMAP A549 cells were transfected with the pEGFP-C1 TIMAP wild-type plasmid encoding human TIMAP, generated previously [26]. Transfections were carried out using Lipofectamine 3000 (Invitrogen Corporation, Carlsbad, CA, USA) according to the manufacturer’s instructions. Protein levels were analysed 48 h post-transfection by Western blot.

### ECIS measurements

Electric cell-substrate impedance sensing (ECIS) was performed using ECIS model Zθ (Applied BioPhysics Inc., Troy, NY) to monitor changes in transepithelial electrical resistance [37]. Cells were seeded onto type 8W10E electrode arrays (10^5^ cells/well). To monitor cell attachment and spreading, impedance was measured at 64 kHz, a frequency sensitive to changes in cell-substrate adhesion. For in vitro wound healing assay, impedance was measured at 4 kHz, which reflects changes in cell-cell contacts. Migration rate was evaluated as previously described in [38]. For the ECIS-based invasion assay, BPAECs were seeded onto 8W10E electrode arrays and allowed to form confluent monolayers. pLKO.1 control and shTIMAP A549 cells (10^5^ cells/well) were gently seeded onto the endothelial layer. Impedance was recorded in at 4 kHz to monitor disruption of the endothelial barrier during cell transmigration, which is reflected as a decrease in resistance and overall impedance.

### SDS-page and western blotting

In all experiment, equal amounts of total protein (30–50 µg) were loaded onto 10–12% SDS-polyacrylamide gels and separated by electrophoresis. Western blotting was performed as described in [39]. The primary and secondary antibodies are listed in Table 1. ImageJ software (version 1.54) was used for densitometry.

**Table 1:** List of antibodies used in Western blot

Antibody	Dilution	Vendor (cat#)
Akt1	1:1000	Cell Signalling #2938S
Anti-Actin	1:1000	Sigma-Aldrich #A5060
Anti-mouse IgG, HRP-linked Antibody	1:5000	Cell Signalling #7076S
Anti-rabbit IgG, HRP-linked Antibody	1:5000	Cell Signalling #7074S
beta-Catenin (D10A8)	1:1000	Cell Signalling #8480
CD24 (SN3)	1:1000	Thermo Fisher Scientific #MA5-11828
CD44	1:1000	Abcam #ab157107
CD133	1:1000	Novus Biologicals a Bio-Techne Brand #NB120-16518
Claudin-1 (D5H1D)	1:1000	Cell Signalling Technology #13,255
E-Cadherin (24E10)	1:1000	Cell Signalling Technology #3195
Human Phospho-Smad2/3 (S465/S467)	1:1000	Bio-Techne R&D System #MAB8935
Lamin A/C (E-1)	1:2000	Santa Cruz Biotechnology #sc-376,248
N-Cadherin (D4R1H)	1:1000	Cell Signalling Technology #13,116
Phospho-β-Catenin (Ser552)	1:1000	Cell Signalling Technology #9566S
Phospho-β-Catenin (Ser675)	1:1000	Cell Signalling Technology #9567S
Phospho-Akt (Ser473)	1:1000	Cell Signalling Technology #4060S
Smad2/3 (C-8)	1:1000	Santa Cruz Biotechnology #sc-133,098
Snail (C15D3)	1:1000	Cell Signalling Technology #3879
Slug (C19G7)	1:1000	Cell Signalling Technology #9585
TIMAP (W-17)	1:1000	Santa Cruz Biotechnology #sc-79,620
ZO-1 (D7D12)	1:1000	Cell Signalling Technology #8193

### Subcellular fractionation

Nuclear fractions from pLKO.1 and sh_16_TIMAP A549 cells were prepared as previously described [40]. The efficiency of the fractionation was assessed by immunoblotting, using actin as a cytoplasmic marker and lamin A/C as a nuclear marker. β-catenin signals were normalized to lamin A/C levels.

### RNA isolation, cDNA synthesis and qPCR

Total RNA was isolated from pLKO.1 control and shTIMAP A549 cells using a GeneJET RNA Purification Kit (Thermo Scientific, Waltham, MA, USA) according to the manufacturer’s instructions. For cDNA synthesis, 2 µg of RNA was reverse transcribed using Maxima Reverse Transcriptase™ (Thermo Scientific, Waltham, MA, USA) with an oligo-d(T)_16_ primer (Promega, Madison, WI, USA). Quantitative real-time PCR analysis was performed on a LightCycler 480 Thermocycler (Roche, Basel, Switzerland, Europe) using Maxima SYBR Green qPCR Master Mix (Thermo Scientific). qPCR was performed with an initial polymerase activation step at 95 °C for 10 min. This was followed by 50 cycles in which denaturation was carried out at 95 °C for 15s, annealing and extension at 60 °C for 30s. Melting curve analysis was conducted by healing the samples to 95 °C for 5 s, followed by cooling to 65 °C for 1 min and then gradually increasing the temperature from 65 °C to 97 °C at a rate of 0.11 °C per second. Three technical replicates were used. GAPDH was used as the reference gene for normalization of Ct values. Relative gene expression levels were determined using the 2^−ΔΔCt^ method as described in [41]. Primer sequences used for amplification are provided in Table 2 according to MIQE guideline [42]. Primers were synthesized by Integrated DNA Technologies (Coralville, IA, USA).

**Table 2:** Primer sequences used for qPCR

Name	Primers (5’-3’)	Amplicon length (bp)	Location	Specificity check	Splice variants targeted
CLDN1 (Claudin-1) NM_021101.5	F: ATGAGGATGGCTGTCATTGG R: ATTGACTGGGGTCATAGGGT	123	Exonic Fw: 586–605 Rev: 708 − 689	BLAST	No
CDH1 (E-cadherin) NM_004360.5	F: GTCTGTAGGAAGGCACAGCC R: TGCAACGTCGTTACGAGTCA	285	Exonic Fw: 2204–2223 Rev: 2488 − 2469	BLAST	No
CDH2 (N-cadherin) NM_001792.5	F: GTGCCATTAGCCAAGGGAATT R: GGAGGAATTCCATTGTCAGAAG	350	Exonic Fw: 1510–1530 Rev: 1859 − 1838	BLAST	No
PPP1R16B (TIMAP) NM_015568.4	F: TGGAGCTAGTCTCAGTGCAAGG R: CTCAAGGATGACTTGTGCCTCAG	147	Exonic Fw: 1049–1070 Rev: 1195 − 1173	BLAST	No
SNAI1 (Snail) NM_005985.4	F: CTGCAGGACTCTAATCCAG R: CAAGGAAGAGACTGAAGTAG	300	Exonic Fw: 144–162 Rev: 443 − 424	BLAST	No
SNAI2 (Slug) NM_003068.5	F: AGATGCATATTCGGACCCACA R: CCTCATGTTTGTGCAGGAGAG	258	Exonic Fw: 688–708 Rev: 945 − 925	BLAST	No

### RNA sequencing (RNA-seq)

Total RNA was isolated from pLKO.1 and sh_16_TIMAP A549 cells as described above. Three biological replicates were prepared for each condition. RNA samples were submitted to Novogene GmbH (Munich, Germany) for quality assessment, library preparation, sequencing and bioinformatic analysis using their standard RNA-seq pipeline. Sequencing was performed on an Illumina platform and read alignment was performed using HISAT2.

### Seahorse extracellular flux analysis

The cellular oxygen consumption rate (OCR) and glycolytic activity were measured using a Seahorse XF96 Extracellular Flux Analyzer (Agilent Technologies, Inc., Santa Clara, CA, USA) [43]. pLKO.1 and shTIMAP A549 cells were seeded into XF96 cell culture microplates (Agilent) and sensor cartridges were hydrated in Seahorse calibration buffer according to the manufacturer’s protocol. The following day, cells were incubated in Seahorse assay medium supplemented with 5% glucose and 1% L-glutamine. Baseline OCR and extracellular acidification rate (ECAR) were recorded by performing five consecutive measurements under untreated conditions. Subsequently, etomoxir (ETO, 50 µM) or oligomycin (OLIGO, 2 µM) were added, followed by five additional measurements. Antimycin (10 µM) was applied to inhibit mitochondrial respiration, and background OCR was measured in five consecutive recordings. All values were normalized to total protein content. Fold changes were determined relative to the corresponding control conditions. Baseline OCR was calculated as basal respiration after subtracting antimycin-resistant respiration. Etomoxir-resistant OCR (etomoxir − antimycin) was interpreted as oxygen consumption derived from glucose and amino acid oxidation, whereas etomoxir-sensitive OCR (baseline − etomoxir) reflected fatty acid oxidation (FAO). Oligomycin-resistant OCR (oligomycin − antimycin) represented uncoupled respiration, and oligomycin-sensitive OCR (baseline − oligomycin) indicated ATP production–linked respiration. The raw data underlying the figures are available on Figshare (DOI: 10.6084/m9.figshare.31177102).

### Sulforhodamine B (SRB) assay

The SRB assay quantified total protein, which correlates with cell number and was measured as an indicator of cell proliferation or to normalize Seahorse extracellular flux measurements [44]. After completion of the Seahorse assay, cells remaining in the XF96 plates were fixed with 10% trichloroacetic acid (TCA) at 4 °C for 1 h and were subsequently washed thoroughly with distilled water. Cells were then stained with 0.4% SRB solution in 1% acetic acid for 10 min at room temperature. Unbound dye was removed by repeated washes with 1% acetic acid, and the bound dye was solubilized in 10mM Tris base. Absorbance was measured at 540 nm using Thermo Scientific Multiskan GO plate reader. The obtained protein values were used for normalization of OCR and ECAR parameters in the Seahorse analysis, ensuring that metabolic parameters reflect differences in cellular activity.

### Cytokine array analysis

Cell culture supernatants were collected from three biological replicates of pLKO.1 and sh_16_TIMAP A549 cells to assess secreted cytokines. Cytokine profiling was performed using the Proteome Profiler Human XL Cytokine Array Kit (R&D Systems, Cat#ARY022B), according to the manufacturer’s instructions. Briefly, the array membranes, pre-spotted with capture antibodies for a wide panel of human cytokines, were activated and incubated with the collected supernatants to allow binding of secreted proteins. After washing steps, membranes were incubated with a cocktail of biotinylated detection antibodies, followed by streptavidin-horseradish peroxidase. Signals were visualized using chemiluminescence and images were captured to evaluate relative cytokine levels. Data from each membrane were quantified by densitometry, with the average intensity calculated for parallel spots corresponding to each cytokine. No signal was detected for the negative control spots.

### Transcriptomic and survival analysis

We examined the expression of PPP1R16B in LUAD using several publicly available datasets. In Gene Expression Profiling Interactive Analysis (GEPIA) database (http://gepia2.cancer-pku.cn/#analysis) [45] the Expression module was applied with the following settings: p-value cutoff of 0.01, LUAD dataset, log_2_(TPM + 1) for log-scale transformation, and matched TCGA normal with GTEx data (n(tumor) = 483, n(normal) = 347). Statistical significance was determined by one-way ANOVA, as provided by the platform (*p* < 0.01). Overall survival of LUAD patients in relation to PPP1R16B expression was also evaluated using GEPIA. Patients were divided into high and low expression quartiles based on median expression values. Kaplan-Meier survival curves were generated, and statistical significance was determined using the log-rank test. Sample sizes were *n* = 239 for the high expression group and *n* = 238 for the low expression group. Disease-free survival (DFS) plot was also generated by GEPIA.

PPP1R16B expression was further assessed using the University of ALabama at Birmingham CANcer data analysis Portal (UALCAN) (https://ualcan.path.uab.edu/) [46, 47]. Expression levels were displayed as box plots (n(primary tumor) = 515, n(normal) = 59) using TCGA Gene module, TCGA datasets for LUAD. Statistical significance was assessed by Student’s t-test. PPP1R16B expression across different cancer stages was analysed using the Individual Cancer Stage module. Expression levels were visualized as box plots (n(normal) = 59, n(stage1) = 277, n(stage2) = 125, n(stage3) = 85, n(stage4) = 28) with statistical significance between stages determined by Student’s t-test. Overall survival was also examined in the Survival module of UALCAN database. Patients were separated into high (*n* = 124) and low (*n* = 378) expression groups. Kaplan-Meier survival curves were generated, and statistical significance was assessed by the log-rank test (*p* = 0.029). Furthermore, a gene signature was derived by intersecting genes positively correlated with PPP1R16B from UALCAN with the set of downregulated genes identified in our RNA-seq analysis, resulting in 11 overlapping genes (ABCB1, ADAP2, CALHM2, CAMK4, COL14A1, FRMD4B, ICOSLG, IGSF10, KIAA0040, SYK, TLR4). Overall survival in LUAD patients was analysed using this gene set in the Survival Analysis model of GEPIA database. Kaplan-Meier survival curves were generated, with statistical significance assessed by the log-rank test.

PPP1R16B expression was also examined using the TNMplot database (https://tnmplot.com/analysis/) [48]. RNA-seq data were queried under the TN plot module in LUAD. Two comparisons were performed: (i) tumor samples versus normal tissues from non-cancerous patients and pediatric controls, (n(tumor) = 524, n(normal) = 486) and (ii) paired tumor and adjacent normal tissues (n(tumor) = 57, n(normal) = 57). Statistical comparisons were automatically calculated by the platform using Mann-Whitney test. Analysis was performed for PPP1R16B expression across different cancer stages in lung tissue from Gene Chip data using the TNM plot module. Expressions were visualized as box plots (n(normal) = 391, n(tumor) = 1865, n(metastatic) = 8) and statistical significance was calculated by Kruskal Wallis test.

Overall survival of LUAD patients based on PPP1R16B (KIAA0823) expression was also analysed using the KM Plotter database (https://kmplot.com/analysis/) [49, 50]. Patients were divided into high and low expression groups according to the median expression. Kaplan-Meier survival curves were generated for all stages (*n* = 1161) or for stage I-III of LUAD (n(stageI) = 370, n(stageII) = 136, n(stageIII) = 24).

All datasets were retrieved on August 26, 2025.

### Statistical analysis

Statistical analyses were performed using GraphPad Prism (version 8.0.1). The specific statistical tests are indicated in the respective figure legends. Significance levels were defined as follows: *p* < 0.05 (*); *p* < 0.01 (**); *p* < 0.001 (***); *p* < 0.0001 (****).

## Results

### TIMAP knockdown alters morphology, cell adhesion and migration of A549 cells

**Fig. 1:**
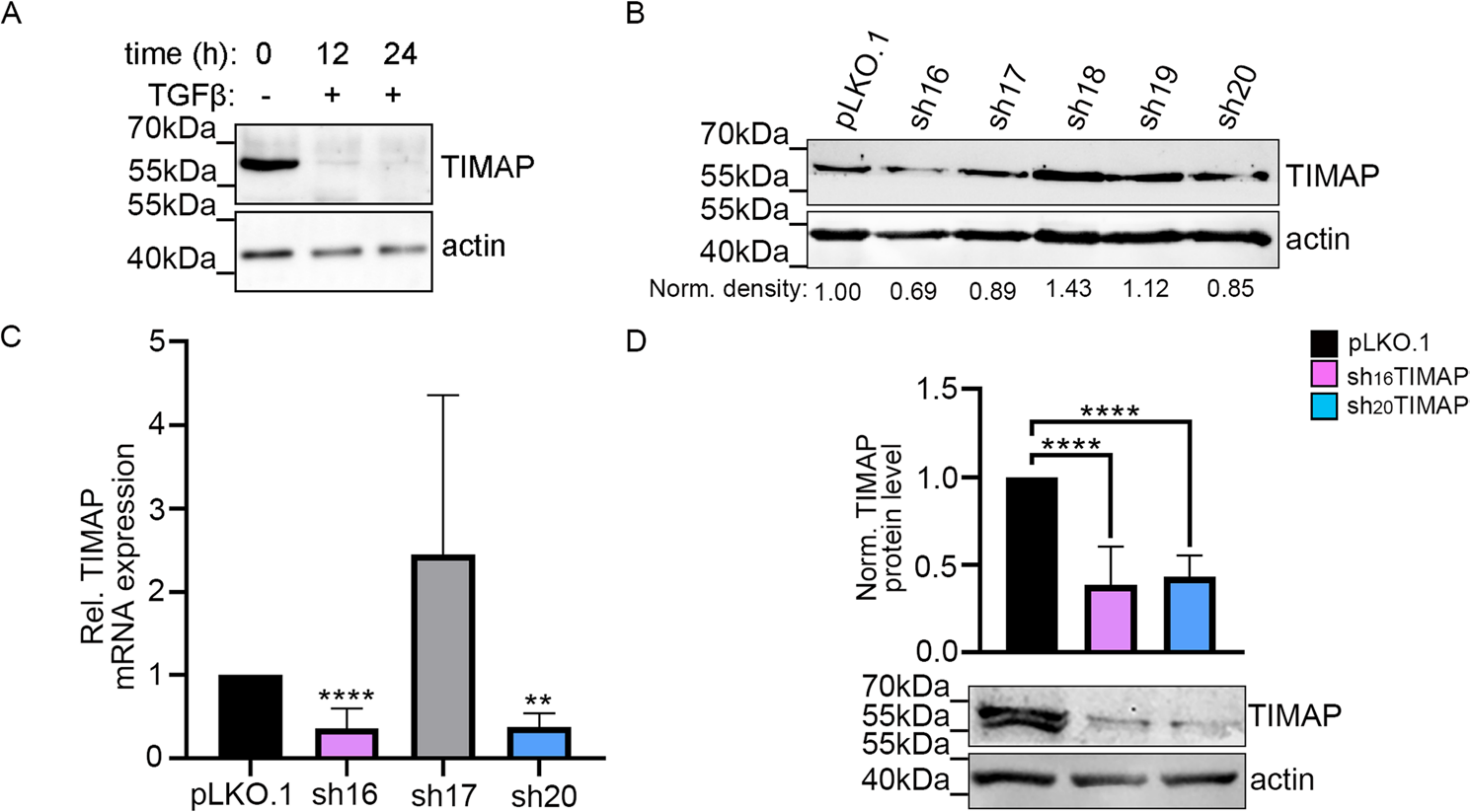
Regulation of TIMAP expression by TGFβ and shRNA-mediated knockdown in A549 cells. **A** Western blot analysis of TIMAP expression in A549 cells treated with 5 ng/ml TGF-β using antibodies against TIMAP and actin. Samples were collected prior to treatment (0 h) and at 12 h and 24 h post-treatment. Actin was used as loading control **B** Five TIMAP depleted (shTIMAP) clones were generated via viral transduction using shRNA constructs targeting different region of the TIMAP transcript. Western blot analysis was performed on pLKO.1 control and TIMAP clones (sh16, sh17, sh18, sh19 and sh20). **C** RT-qPCR was performed on pLKO.1 control and TIMAP clones sh16, sh17 and sh20, confirming a significant reduction in TIMAP mRNA levels (*n* = 3–12, mean ± SD, ** *p* < 0.01 ****p < 0.0001). TIMAP expression was normalized to GAPDH and actin as controls. **D** Western blot analysis of TIMAP expression in pLKO.1 and shTIMAP A549 cell lysates (sh16 and sh20 clones). Actin was used as loading control for normalization. Statistical significance was carried out using one-way ANOVA (*n* = 4–12, ****p < 0.0001)

**Fig. 2:**
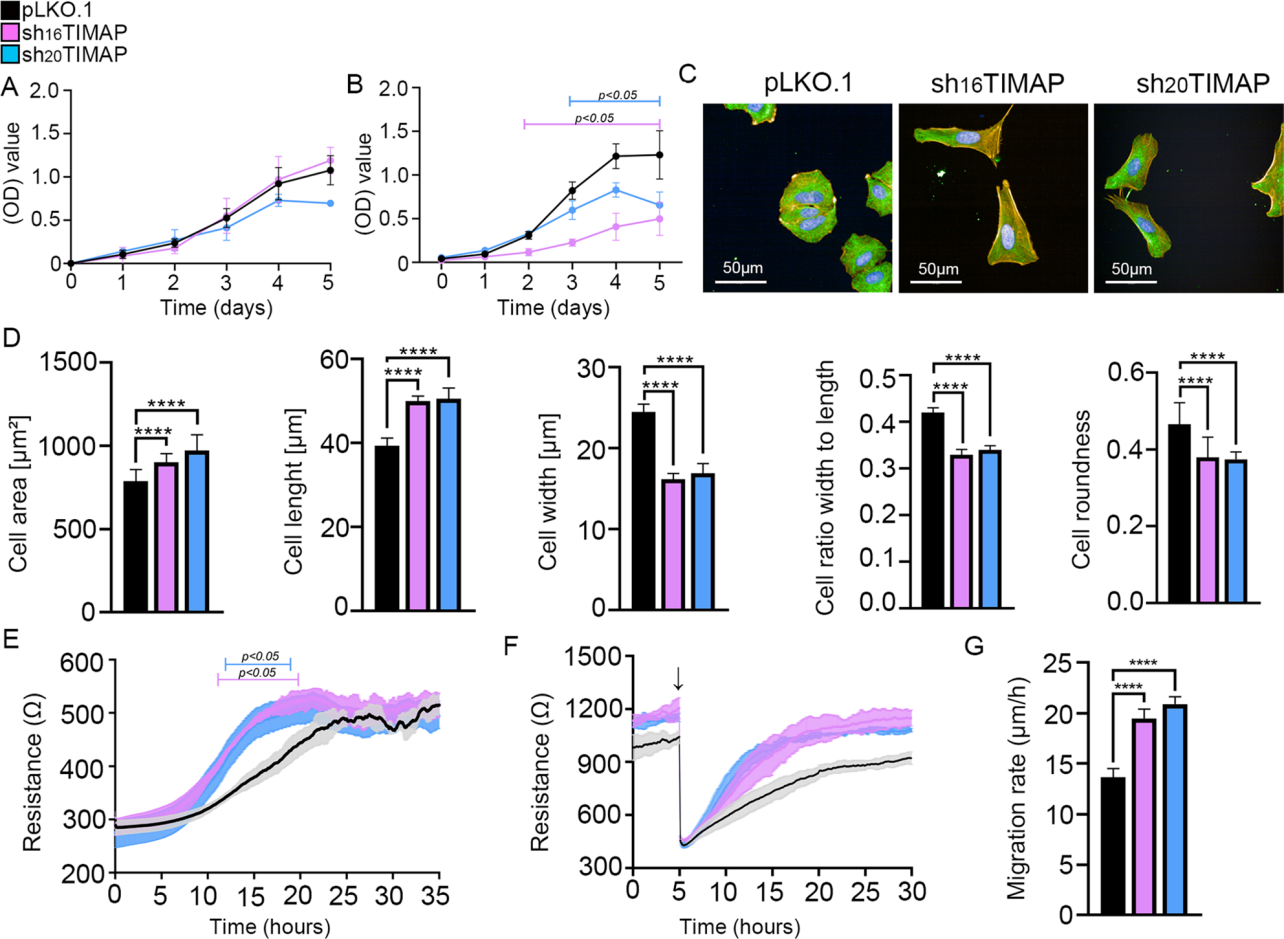
TIMAP depletion in A549 cells alters cell morphology, adhesion and motility. ** A** Cell viability was measured by MTT assay at 540 nm (*n* = 3–6, mean ± SD). **B** Proliferation of pLKO.1 and shTIMAP cells was measured by SRB assay at 540 nm. Statistical analysis was performed using unpaired t-test, *n* = 7–10, *p < 0.05). **C** Representative images of sh_16_TIMAP, sh_20_TIMAP and pLKO.1 control cells were acquired using the Opera Phenix High Content Screening system. Cells were stained with Texas Red phalloidin (red), tubulin (green) and DAPI (nuclei, blue). Scale bar: 50 μm. **D** Quantitative analysis of cell shape parameters were performed using build-in Harmony Software. Statistical analysis was performed using an one-way ANOVA on more than 10,000 cells were analysed per group (****p < 0.0001). **E** Cell spreading and attachment were monitored using ECIS. Statistical analysis was carried out using multiple t-tests (*n* = 9,*p < 0.05, mean ± SD) **F** An in vitro wound healing assay was performed using ECIS. A high current pulse (indicated by the arrow) was applied to a confluent monolayer to create a wound. Resistance was continuously monitored (*n* = 9, mean ± SD). Error bars indicate standard deviation. **G** Statistical significance between control and TIMAP-silenced cell migration rate was assessed using one-way ANOVA (****p < 0.0001)

Downregulation of TIMAP (PPP1R16B; UNIPROT: Q96T49) upon TGF-β1 treatment has previously been reported in endothelial cells [25]. To assess whether this regulation also occurs in carcinoma, we used human adenocarcinoma A549 cell line as a model system. Treatment with TGF-β1 for 12 and 24 h reduced TIMAP protein expression, indicating that TIMAP is responsive to TGF-β1 in this carcinoma model (Fig. 1A). To achieve stable depletion of TIMAP, we utilized a lentiviral shRNA-mediated system. Following transduction, cells were selected using puromycin to ensure the integration. Five different shRNA sequences targeting TIMAP were initially tested. Based on the preliminary Western blot results (Fig. 1B), three shRNAs (sh16, sh17, sh20) were further evaluated by RT-qPCR (Fig. 1C). TRCN0000006916 (sh16) and TRCN0000006920 (sh20) exhibited the highest knockdown efficiency and were therefore selected for subsequent experiments. The resulting cell lines are hereafter referred to as sh_16_TIMAP and sh_20_TIMAP, respectively. The decrease in TIMAP mRNA level in shTIMAP cells was accompanied by a significant reduction in TIMAP protein levels as confirmed by Western blots (Fig. 1D). These findings confirm a successful knockdown of TIMAP expression in the A549 cell line at both transcript and protein levels. To assess whether TIMAP depletion affects cell viability and proliferation, MTT (Fig. 2A) and SRB assays (Fig. 2B) were performed. While MTT assay results indicated no change in the rate of spontaneous apoptosis, SRB measurements revealed a significant difference in both shTIMAP cells compared to control cells. These results suggest that depletion of TIMAP exerts a cytostatic rather that cytotoxic effect. Upon TIMAP depletion, HCS imaging revealed significant changes in cell morphology as shTIMAP cells exhibited a larger, more elongated phenotype compared to pLKO.1 control cells (Fig. 2C). Quantitative measurements confirmed a significant increase in cell area and length, along with reduced width, roundness and width to length ratio, which are characteristic of a mesenchymal phenotype (Fig. 2D). These data suggest that TIMAP depletion induces a morphological shift toward mesenchymal characteristics. As mesenchymal phenotype is often associated with enhanced motility, therefore we evaluated cell attachment and spreading using ECIS. After seeding, both shTIMAP cell line showed a more rapid increase in impedance compared to pLKO.1 control cells, indicating enhanced attachment (Fig. 2E). To study cell migration, we performed a wound healing assay using ECIS. Confluent cell monolayers were subjected to a brief high voltage pulse to create a controlled disruption or “wound” in the cell layer. This disruption caused a drop in resistance as indicated by an arrow and the migration of cells into the wounded “void” area was monitored in real time (Fig. 2F). Both shTIMAP cell lines restored resistance more rapidly than control cells, indicating a higher migration rate and faster migration speed (Fig. 2G).

### Loss of TIMAP induces gene expression changes associated with EMT

**Fig. 3:**
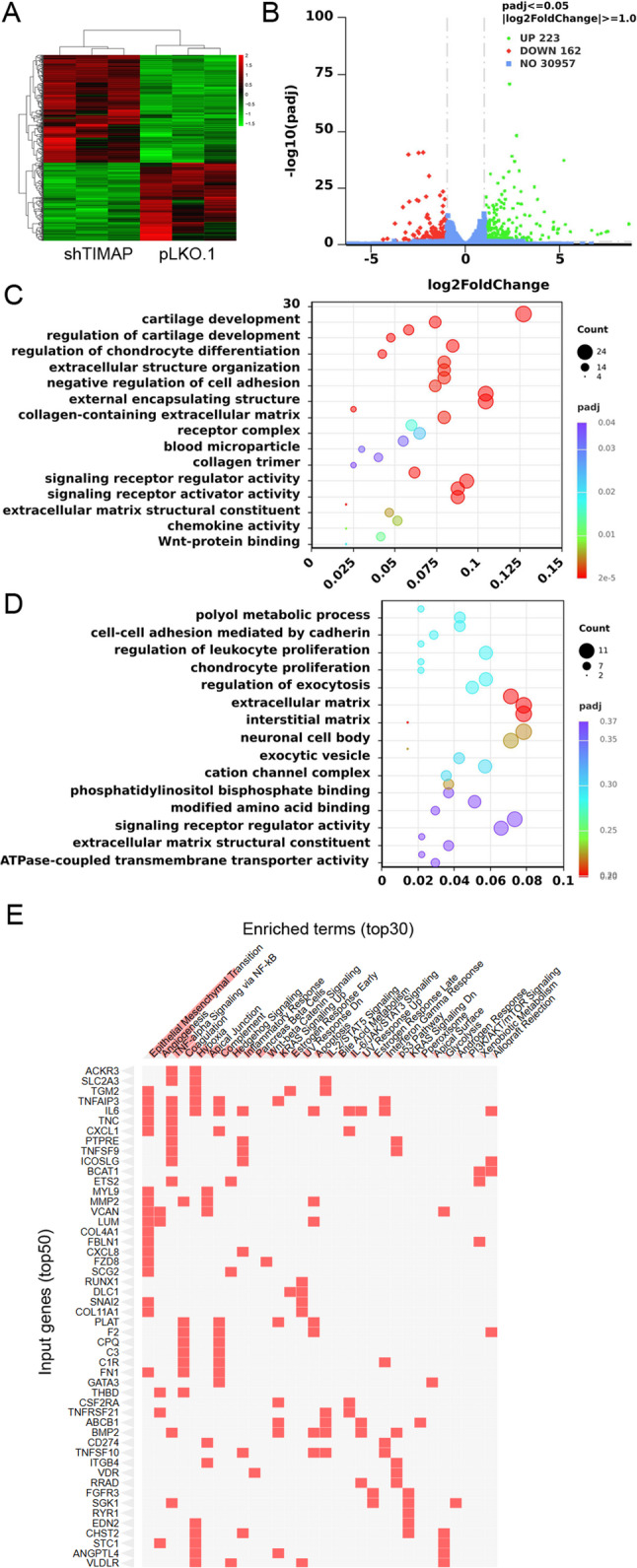
Transcriptomic changes upon TIMAP depletion in A549 cells.** A** Heatmap showing DEGs between TIMAP-depleted cells (shTIMAP) and control (pLKO.1) **B** Volcano plot of DEG, highlighting 223 upregulated (green) and 162 downregulated (red) genes. Blue dots (30957) did not meet the significance or fold change thresholds. **C**,** D** Gene Ontology (GO) enrichment analysis of upregulated (**C**) and downregulated (**D**) genes, with bubble size indicating gene count and color indicating adjusted p-values. **E** Clustergram from Enrichr analysis (MSigDB 2020) of TIMAP-depleted cells, showing top enriched pathways ranked by *p*-value. (Abbreviations: ACKR3: atypical chemokine receptor 3, SLC2A3: solute carrier family 2 member 3, TGM2: transglutaminase 2, TNFAIP3: TNF alpha induced protein 3, IL6: interleukin 6, TNC: tenascin C, CXCL1: C-X-C motif chemokine ligand 1, PTPRE: protein tyrosine phosphatase, receptor type E, TNFSF9: TNF superfamily member 9, ICOSLG: inducible T cell costimulator ligand, BCAT1: branched chain amino acid transaminase 1, ETS2: ETS proto-oncogene 2, transcription factor, MYL9: myosin light chain 9, MMP2: matrix metallopeptidase 2, VCAN: versican, LUM: lumican, COL4A1: collagen type IV alpha 1 chain, FBLN1: fibulin 1, CXCL8: C-X-C motif chemokine ligand 8, FZD8: frizzled class receptor 8, SCG2: secretogranin II, RUNX1: runt related transcription factor 1, DLC1: DLC1 Rho GTPase activating protein, SNAI2: snail family transcriptional repressor 2, COL11A1 : collagen type XI alpha 1 chain, PLAT: plasminogen activator, tissue type, F2: coagulation factor II, thrombin, CPQ: carboxypeptidase Q, C3: complement C3, C1R: complement C1r, FN1: fibronectin 1, GATA3: GATA binding protein 3, THBD: thrombomodulin, CSF2RA: colony stimulating factor 2 receptor alpha subunit, TNFRSF21: TNF receptor superfamily member 21, ABCB1: ATP binding cassette subfamily B member 1, BMP2: bone morphogenetic protein 2, CD274: CD274 molecule, TNFSF10: TNF superfamily member 10, ITGB4: integrin subunit beta 4, VDR: vitamin D receptor, RRAD: Ras related glycolysis inhibitor and calcium channel regulator, FGFR3: fibroblast growth factor receptor 3, SGK1: serum/glucocorticoid regulated kinase 1, RYR1: ryanodine receptor 1, EDN2: endothelin 2, CHST2: carbohydrate sulfotransferase 2, STC1: stanniocalcin 1, ANGPTL4: angiopoietin like 4, VLDR: very low density lipoprotein receptor)

To gain deeper insight into the molecular changes induced by TIMAP depletion, RNA sequencing was performed from the TIMAP-silenced cell line (sh_16_TIMAP) and the non-targeting sequence control. To comprehensively characterize the transcriptional changes induced by TIMAP depletion, we first generated a cluster heat map of all differentially expressed genes (DEGs) to visualize patterns of gene expression across samples (Fig. 3A). Among the analysed genes, 223 were significantly upregulated (log₂ fold change of at least 1 and an adjusted p-value of 0.05 or less), and 162 were significantly downregulated (log₂ fold change of − 1 or less and an adjusted p-value of 0.05 or less), as highlighted by the green and red markers, respectively (Fig. 3B). The remaining genes (blue dots) did not meet the significance or fold change thresholds. These DEGs were subsequently used for further functional validation and pathway analysis to elucidate the role of TIMAP in cellular processes. To investigate the biological processes and molecular functions affected by TIMAP depletion, we performed GO enrichment analysis on significantly upregulated and downregulated genes. GO enrichment analysis of upregulated genes highlighted terms associated with extracellular matrix organization, collagen-containing extracellular matrix, receptor complex formation, and signaling receptor regulator activity (Fig. 3C). Notably, several of these terms such as extracellular matrix organization, negative regulation of cell adhesion, and Wnt-protein binding are well-established hallmarks of EMT. Downregulated genes were enriched for processes including cell-cell adhesion mediated by cadherin, extracellular matrix structural constituent activity, and regulation of leukocyte proliferation (Fig. 3D). The loss of cadherin-mediated adhesion is a canonical EMT feature, further supporting an EMT-associated transcriptional shift upon TIMAP knockdown. Pathway enrichment mapping reinforced that observation that TIMAP depletion induces a transcriptional program closely associated with EMT (Fig. 3E). The DEGs resulting from TIMAP knockdown showed substantial overlap with pathways directly linked to EMT and EMT-associated processes, including angiogenesis, TNF-α/NF-κB signaling, coagulation, and hypoxia. Several of these genes, such as VCAN, LUM, THBS1, TNFAIP3, CXCL1, and MMP2 are well-established contributors to EMT and extracellular matrix remodelling. At the transcriptional level, TIMAP knockdown resulted in hallmark EMT-associated shifts. Epithelial markers, including cadherin-19 (CDH19) and claudin-3 (CLDN3), were significantly downregulated, whereas mesenchymal markers such as MMP2 and fibronectin-1 (FN1), along with EMT-inducing transcription factors (SNAI1, SNAI2, ZEB2), were upregulated.

### TIMAP depletion affects the protein and mRNA expression of EMT markers

**Fig. 4:**
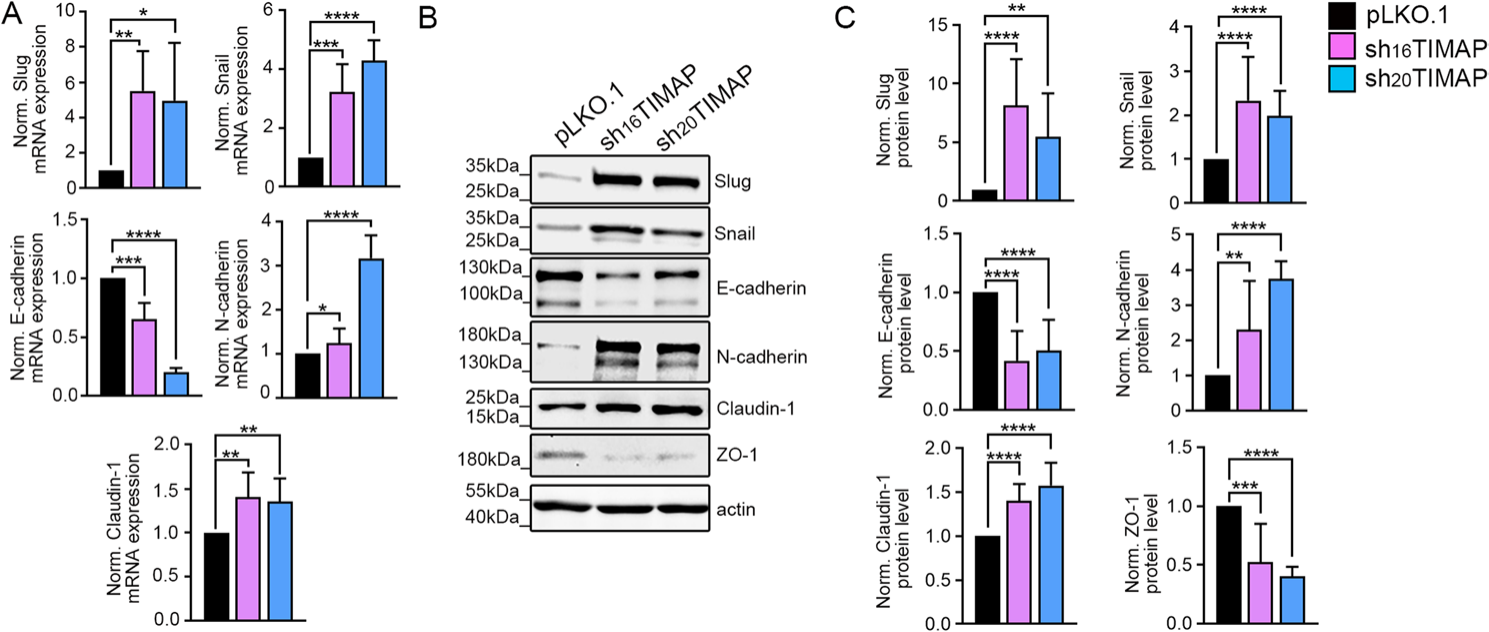
TIMAP depletion alters the protein and mRNA expression of EMT markers.** A** RT-qPCR was performed on pLKO.1, sh_16_TIMAP and sh_20_TIMAP A549 cells. Expression levels were normalized to GAPDH and actin. Statistical analysis was performed using one-way ANOVA (*n* = 3–14, ****p < 0.0001, ***p < 0.001, **p < 0.01 and *p < 0.05). **B** Cell lysates from pLKO.1, sh_16_TIMAP and sh_20_TIMAP A549 cells were analysed by Western blot using antibodies against EMT markers. **C** Densitometric quantification was performed and normalized to actin as a loading control. Statistical significance was determined using one-way ANOVA (*n* = 4–10, ****p < 0.0001, ***p < 0.001, **p < 0.01

To further investigate the effect of TIMAP depletion on EMT, we analysed the expression of classical EMT markers at both mRNA (Fig. 4A) and protein levels (Fig. 4B and C). Transcription factors Snail (SNAI1) and Slug (SNAI2) were significantly upregulated at both mRNA and protein levels, consistent with the activation of EMT in both sh_16_TIMAP and sh_20_TIMAP cell lines. E-cadherin expression was consistently downregulated in shTIMAP cells at both the mRNA and protein levels, while N-cadherin was upregulated, indicating the cadherin switch characteristic of EMT. Interestingly, claudin-1, a tight junction protein associated with epithelial integrity was upregulated at both the mRNA and protein levels. However, the reduction of ZO-1 protein further supports disruption of tight junctions. To assess specificity of TIMAP depletion induced effects, we extended our analysis to the SK-LU-1 lung adenocarcinoma cell line. Stable TIMAP knockdown was generated in SK-LU-1 cells using the same shRNA constructs applied in A549 cells. Although the reduction of TIMAP protein level was less efficient in SK-LU-1 cells compared with A549 cells, TIMAP depletion reproducibly induces protein expression changes characteristic of EMT (Suppl. Figure 1 A). Increased expression of Snail and Slug was observed, together with a cadherin switch, consistent with the EMT-like phenotype identified in A549 cells (Suppl. Figure 1B). Taken together, these results suggest that TIMAP depletion is associated with key features of an EMT phenotype, including repression of epithelial markers, activation of EMT-related transcription factors, and alteration of junctional components.

### TIMAP depletion alters key signalling pathways associated with EMT

**Fig. 5:**
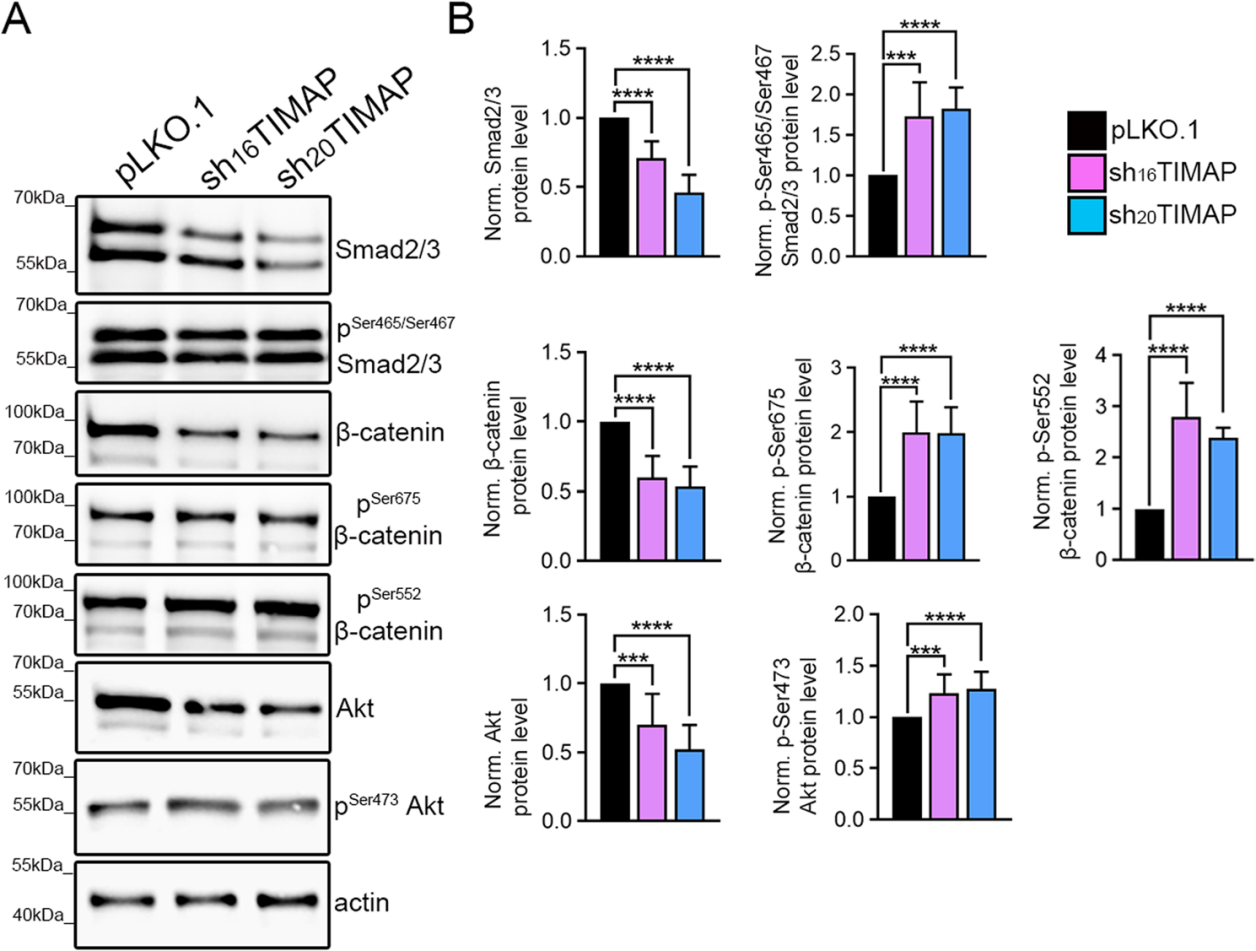
Signaling pathways associated with EMT following TIMAP depletion. **A** Cell lysates of pLKO.1, sh_16_TIMAP and sh_20_TIMAP A549 were analysed by Western blot using antibodies against SMAD2/3, p-Ser465/Ser467 SMAD2/3, β-catenin, p-Ser552 β-catenin, p-Ser675 β-catenin, Akt and p-Ser473 Akt. **B** Total protein levels were normalized to actin and phosphorylation levels to corresponding total protein. Data are presented as mean ± SD (one-way ANOVA, *n* = 5–12, ****p < 0.0001, ***p < 0.001)

**Fig. 6:**
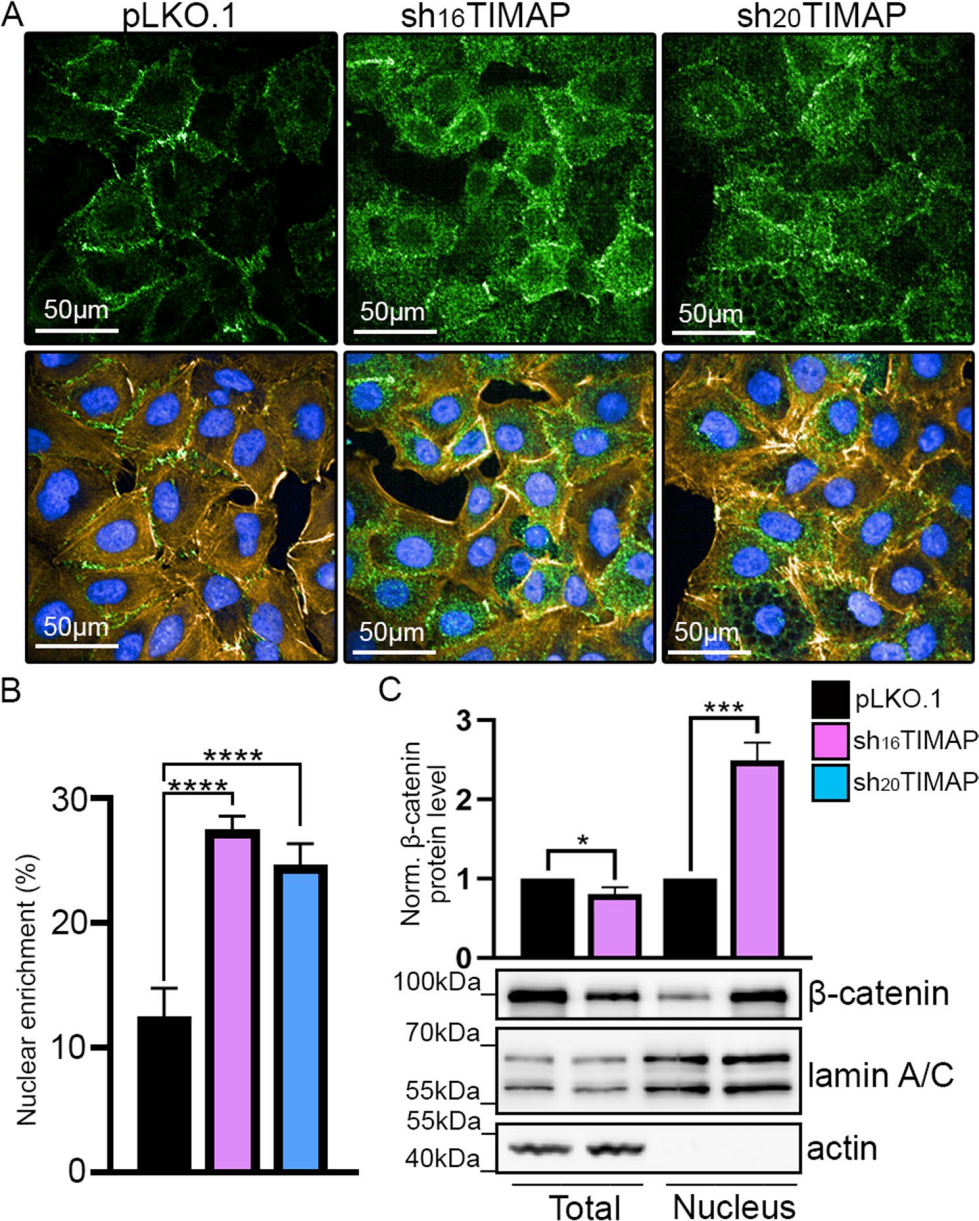
TIMAP depletion induces nuclear translocation of β-catenin.** A** Representative confocal images of pLKO.1, sh_16_TIMAP and sh_20_TIMAP acquired using the Opera Phenix High Content Screening system. Cells were stained with Texas Red-phalloidin (actin, orange), β-catenin (green) and DAPI (nuclei, blue). Scale bars: 50 μm. **B** Quantitative analysis of β-catenin nuclear enrichment calculated using build-in Harmony Software and expressed as percent of nuclear to total β-catenin staining intensity. 1700–1900 cells were analyzed per well. Statistical analysis was performed using one-way ANOVA (*n* = 9, ****p < 0.0001). **C** Subcellular fractionation was performed on pLKO.1 control and sh_16_TIMAP A549 cells. Total cell lysates and nuclear fractions were analysed by Western blot using antibodies against β-catenin, lamin A/C and actin. β-catenin signals were normalized to lamin A/C as a nuclear loading control. Statistical significance was determined using unpaired t-test (*n* = 3, *** *p* < 0.001, *p < 0.05)

To investigate the molecular pathways involved in TIMAP-mediated regulation of EMT, the expression and phosphorylation status of key EMT-related signalling proteins was analysed by Western blot in A549 cells (Fig. 5A). First, we tested the primary intracellular effectors of TGF-β signalling, the SMAD proteins. Phosphorylation of SMAD2/3 at Ser 465/467 is required for their activation, allowing them to form complex with SMAD4 and translocate into the nucleus to regulate the transcription of target genes [51]. In shTIMAP cells, total SMAD2/3 protein levels decreased while phosphorylation of SMAD2/3 (Ser 465/467) were elevated, indicating the activation of the pathway (Fig. 5B). Next, we analysed the Wnt/β-catenin signalling, which is known to promote cell migration and invasion during EMT [52]. Phosphorylation of β-catenin at Ser552 and Ser675 was significantly increased in shTIMAP cells (Fig. 5B), which is associated with enhanced nuclear translocation and transcriptional activity, leading to the upregulation of genes that promote cell growth, proliferation and motility [53]. This indicates that TIMAP depletion may promote EMT through the activation of β-catenin, despite a reduction in total β-catenin levels. Given that kinases such as Akt and protein kinase A (PKA) can phosphorylate β-catenin at Ser552 and on Ser675, respectively [54], we examined Akt activation by analysing its phosphorylation at Ser473 [55]. In shTIMAP cells, phosphorylation of Akt at Ser473 was increased despite the reduction in the total Akt protein level, indicating a strong Akt activity (Fig. 5B). Since phosphorylation of Akt at Ser473 is predominantly mediated by mammalian target of rapamycin complex 2 (mTORC2) [56], these findings indicate elevated mTORC2 activity in the absence of TIMAP. The observed increase in β-catenin phosphorylation accompanied by a reduction in total β-catenin protein levels suggested enhanced nuclear accumulation [53]. To investigate this, β-catenin localization was analysed by confocal HCS. Cells were immunostained with a β-catenin specific antibody, while F-actin was visualized using Texas Red-conjugated phalloidin (Fig. 6A). In control cells, β-catenin displayed prominent membrane localization, whereas in sh_16_TIMAP and sh_20_TIMAP cells, β-catenin exhibited a more diffuse distribution, including nuclear staining. Quantitative analysis using the build-in Harmony software assessed β-catenin nuclear accumulation based on nuclear-to-total staining intensity ratio and revealed a significant increase in nuclear β-catenin enrichment in TIMAP-depleted cells (Fig. 6B). Consistent with these findings, nuclear fraction isolated from control and sh_16_TIMAP cells demonstrated increased nuclear β-catenin level in TIMAP depleted cells by Western blot analysis (Fig. 6C).

Together, these results suggest that TIMAP depletion activates multiple signaling pathways in A549 cells, including TGF-β/SMAD and Wnt/β-catenin, as well as mTORC2/Akt, which collectively promote EMT characteristics, such as migration, invasion and proliferation.

### Re-expression of TIMAP or TGF-β pathway inhibition suppresses EMT-related signaling

**Fig. 7:**
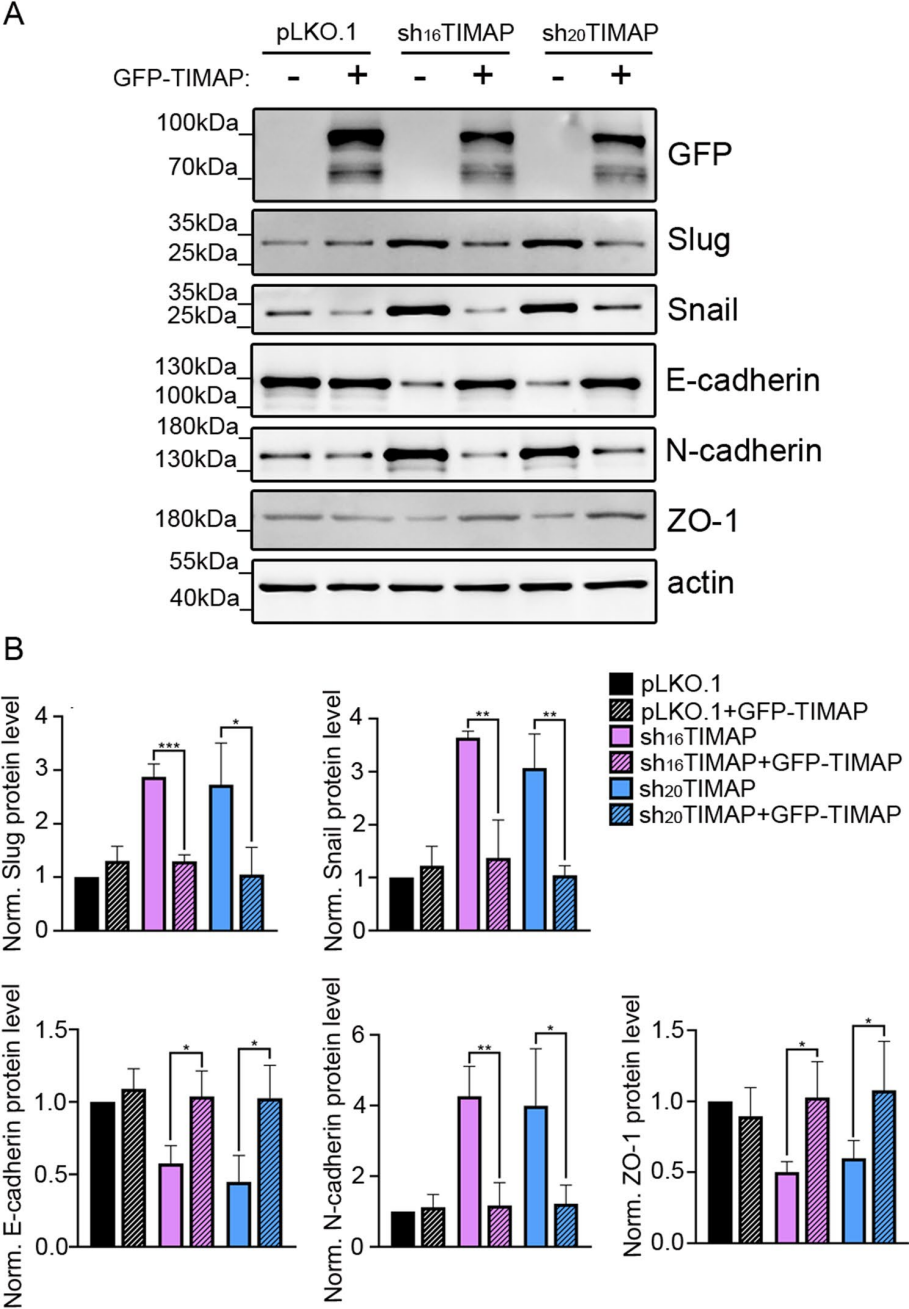
Re-expression of TIMAP reverses EMT-associated changes.** A** pLKO.1, sh_16_TIMAP and sh_20_TIMAP A549 cells were transfected with GFP-tagged TIMAP expression plasmid. Cell lysates from control and GFP-TIMAP transfected cells were analyzed by Western blot using anti-GFP antibody to detect recombinant TIMAP and antibodies against EMT markers. **B** Densitometric analysis was performed and normalized to actin as a loading control. Statistical significance was determined using one-way ANOVA (*n* = 3, ***p < 0.001, **p < 0.01, *p < 0.05)

To examine whether re-expression of TIMAP influences EMT-associated changes, pLKO.1 or shTIMAP A549 cells were transfected with a pEGFP-C1 TIMAP wild-type construct (GFP-TIMAP), followed by analysis of EMT markers. Overexpression of recombinant TIMAP was confirmed by Western blot using GFP-specific antibody (Fig. 7A). In pLKO.1 controll cells, overexpression of GFP-TIMAP did not significantly altered the levels of EMT marker proteins (Fig. 7B). In contrast, in shTIMAP cells with reduced endogenous TIMAP levels, expression of GFP-TIMAP was associated with decreased levels of the EMT-associated transcription factors Snail and Slug to levels comparable with pLKO.1 controls. Moreover, ZO-1 and cadherin expressions were restored, with E-cadherin increased and N-cadherin decreased, indicating a shift back toward an epithelial phenotype (Fig. 7B). These results suggest that re-expression of TIMAP in shTIMAP cells partially counteracts EMT-associated molecular changes, consistent with a potential negative regulatory role of TIMAP in EMT.

**Fig. 8:**
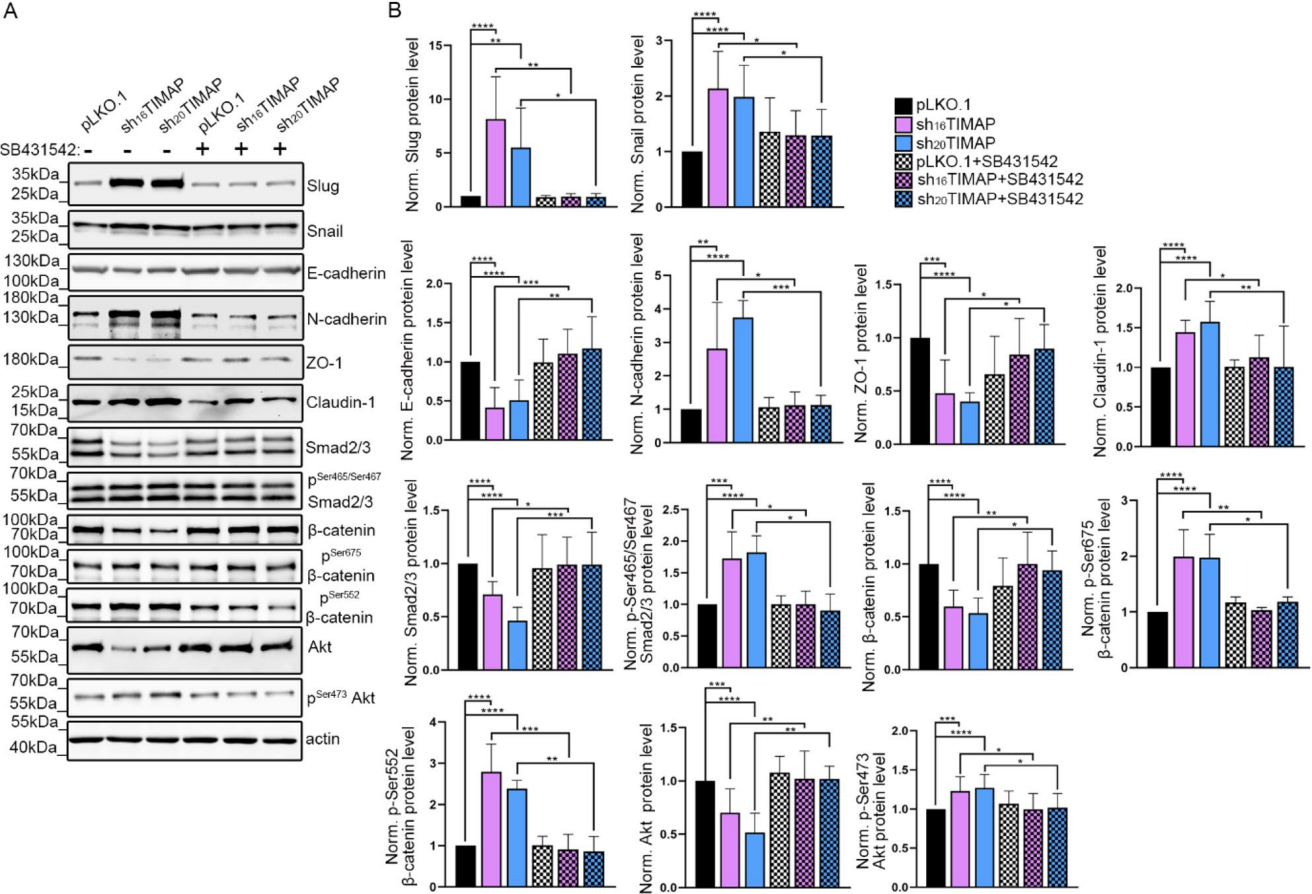
EMT-like changes induced by TIMAP depletion are dependent on TGF-β signaling.** A** Western blot analysis of pLKO.1 control, sh_16_TIMAP and sh_20_TIMAP A549 cells treated with or without the ALK5 inhibitor SB-431542 (10 µM, 48 h). Cell lysates were analysed using antibodies against EMT markers. Actin was used as a loading control. **B** Densitometric quantification of protein expression and phosphorylation levels normalized to actin or the corresponding total protein. Data are presented as mean ± SD. Statistical analysis was performed using one-way ANOVA (****p < 0.0001, ***p < 0.001, **p < 0.01, *p < 0.05)

To determine whether the EMT-like changes observed in shTIMAP cells were dependent on TGF-β signaling, we treated the cells with the selective TGF-β type I receptor (ALK5) inhibitor SB-431542 [57] (Fig. 8A). Treatment of shTIMAP A549 cells with 10 µM SB-431542 for 48 h prevented the upregulation of Snail and Slug and restored cadherin expression levels (Fig. 8B). Moreover, treatment of shTIMAP cells with SB-431542 not only reversed the EMT marker changes but also normalized key signaling pathway activations. Inhibition of TGF-β signaling with SB-431542 restored the total protein levels of SMAD2/3, β-catenin and Akt toward control levels and substantially reduced their phosphorylation, thereby decreasing pathway activation. These results demonstrate that the enhanced activation of SMAD2/3, Akt or β-catenin observed in TIMAP-depleted cells is dependent on TGF-β signaling.

### TIMAP depletion disrupts 3D spheroid integrity and enhances invasive behaviour

**Fig. 9:**
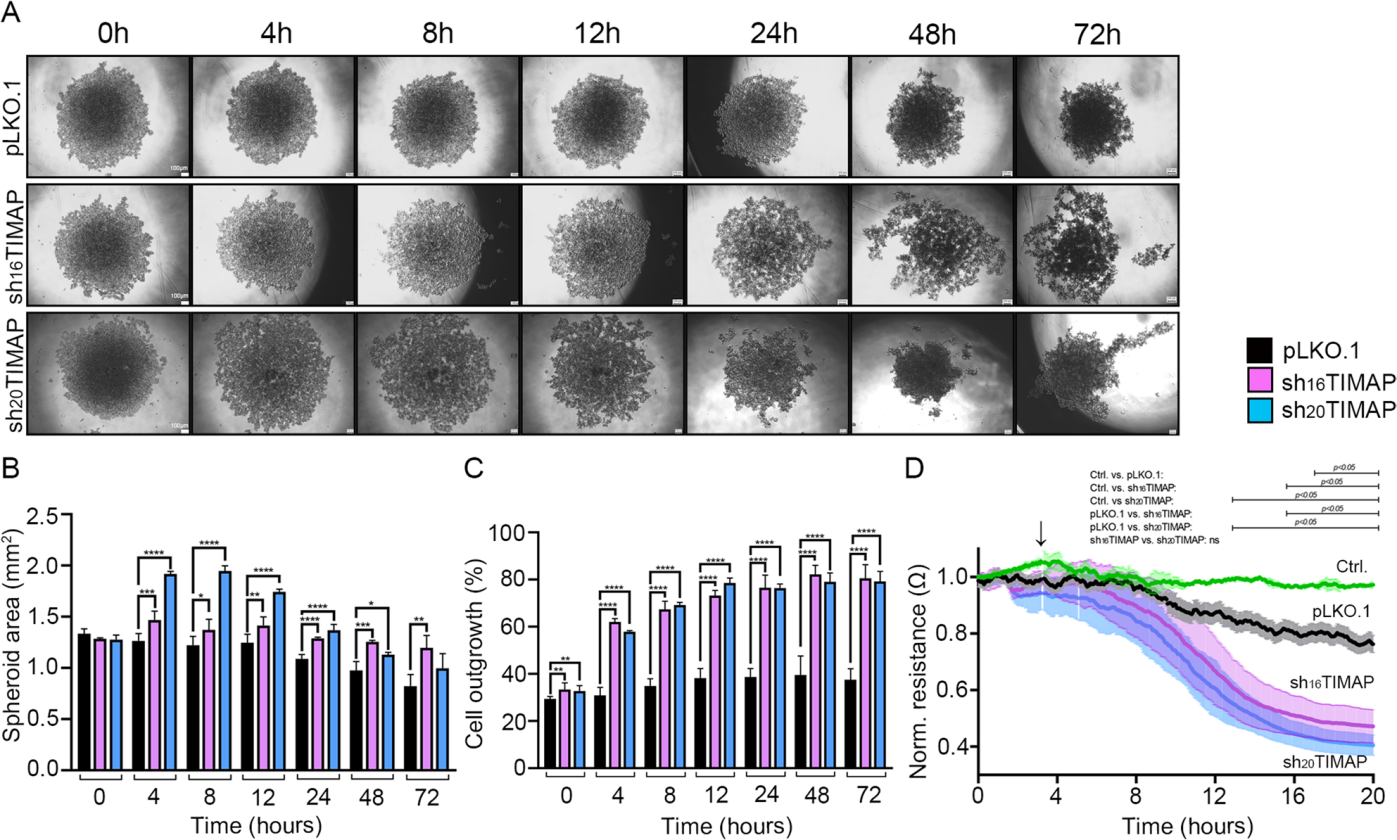
Silencing of TIMAP induces excursion of cells from spheroids.** A** Representative images of spheroids formed by pLKO.1 control, sh_16_TIMAP and sh_20_TIMAP A549 cells. Scale bar: 100 μm. **B** Spheroid size was quantified using ImageJ. and statistically analysed using one-way ANOVA (*n* = 3–8, ****p < 0.0001, ***p < 0.001, **p < 0.01 and *p < 0.05) **C** Spheroid fragmentation (%) was determined by calculating the ratio of the area of released cells to the total spheroid area, multiplied by 100. Statistical analysis was done using unpaired t-test (*n* = 3–8, **p < 0.01, ****p < 0.0001). **D** ECIS-based invasion assay was made by seeding pLKO.1 (black), sh_16_TIMAP (purple) and sh_20_TIMAP (blue) cells onto confluent monolayer of BPAEC, indicated by the arrow. Green line represents BPAEC cells alone, as control. Statistical analysis was performed using two-way ANOVA (*n* = 3, *p* < 0.05)

As cancer cells within spheroids can invade into the surrounding matrix, a 3D spheroid assay was performed to assess the effect of TIMAP depletion in A549 cells. Control pLKO.1 cells formed compact and well-defined spheroids with defined borders, whereas spheroids from TIMAP-depleted cells appeared loosely organized and structurally unstable (Fig. 9A). Moreover, shTIMAP spheroids were significantly larger in size (Fig. 9B), and over time, exhibited pronounced cell detachment from the core compared to the pLKO.1 control spheroids (Fig. 9C). Since cells undergoing EMT are more likely to dissociate and migrate from the spheroid, we assessed invasive capacity using an ECIS-based trans-endothelial migration assay. Control and shTIMAP cells were seeded onto a confluent monolayer of endothelial cells and barrier disruption was followed in time. Barrier disruption was indicated by a decrease in transendothelial electrical resistance values. Compared to control cells, shTIMAP cells induced a more pronounced decrease in resistance, indicating enhanced monolayer disruption and increased invasive potential (Fig. 9D). These results suggest that TIMAP depletion promotes a more invasive EMT-like phenotype, characterized by reduced structural stability and enhanced cell detachment and increased invasiveness.

### TIMAP depletion alters cytokine secretion profile with enrichment of EMT-associated factors

**Fig. 10:**
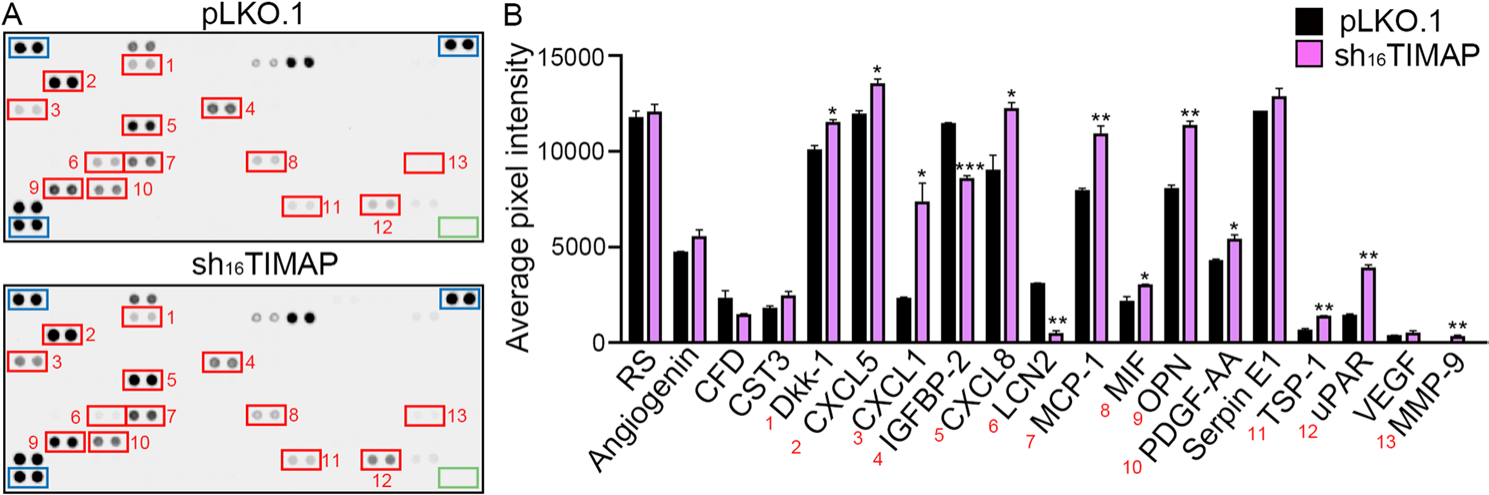
TIMAP depletion induces the expression of pro-EMT cytokines. **A** Representative cytokine array membranes are shown. Red boxes indicate cytokines with significantly altered expression in sh_16_TIMAP cells compared to pLKO.1 control, numbered as in panel B. Reference spots (positive controls) are marked with blue boxes, negative controls with green boxes. **B** Quantification of differentially expressed cytokines based on average pixel intensity, using unpaired t-test. Cytokines with significant expression changes are marked (*p < 0.05, **p < 0.01). (Abbreviations: RS: Reference Spot, CFD: Complement Factor D, CST3: Cystatin C, Dkk-1: Dickkopf-1, CXCL5: ENA-78, CXCL1: GROα, IGFBP: Insulin-like Growth Factor Binding Protein 2, CXCL8: Interleukin-8, LCN2: Lipocalin-2, MCP-1: Monocyte Chemoattractant Protein-1, MIF: Macrophage Migration Inhibitory Factor, OPN: Osteopontin, PDGF-AA: Platelet-Derived Growth Factor AA, TSP-1: Thrombospondin-1, uPAR: Urokinase Plasminogen Activator Receptor, VEGF: Vascular Endothelial Growth Factor, MMP-9: Matrix Metalloproteinase-9)

As cytokines play a major role in EMT and they themselves represents potential therapeutic targets [58], next we aimed to determine how TIMAP depletion affects the cytokine secretory profile of A549 cells. Conditioned media of pLKO.1 control and sh_16_TIMAP A549 cells were compared using a membrane-based cytokine array (Fig. 10A). Densitometric analysis revealed distinct alterations in cytokine secretion following TIMAP depletion. Several cytokines and secreted factors associated with EMT, inflammation and tumor progression were significantly upregulated in sh_16_TIMAP cells such as Dkk-1, CXCL5, CXCL1, CXCL8 (IL-8), MCP-1 (CCL2), MIF, OPN, PDGF-AA, TSP-1, uPAR and MMP-9 (Fig. 10B). In contrast, levels of lipocalin-2 (LCN2) and insulin like growth factor binding protein 2 (IGFBP-2) were reduced in the supernatant of shTIMAP cells. The secretome of shTIMAP cells showed a pro-tumorigenic and pro-inflammatory profile, with upregulation of factors known to drive EMT, angiogenesis and metastatic potential [59–61]. This altered cytokine profile points to the establishment of a microenvironment that supports EMT and tumor progression. We therefore examined whether these changes were also associated with modifications in the metabolic activity of shTIMAP cells.

### TIMAP knockdown is associated with a metabolic shift in A549 cells from oxidative phosphorylation to glycolysis

**Fig. 11:**
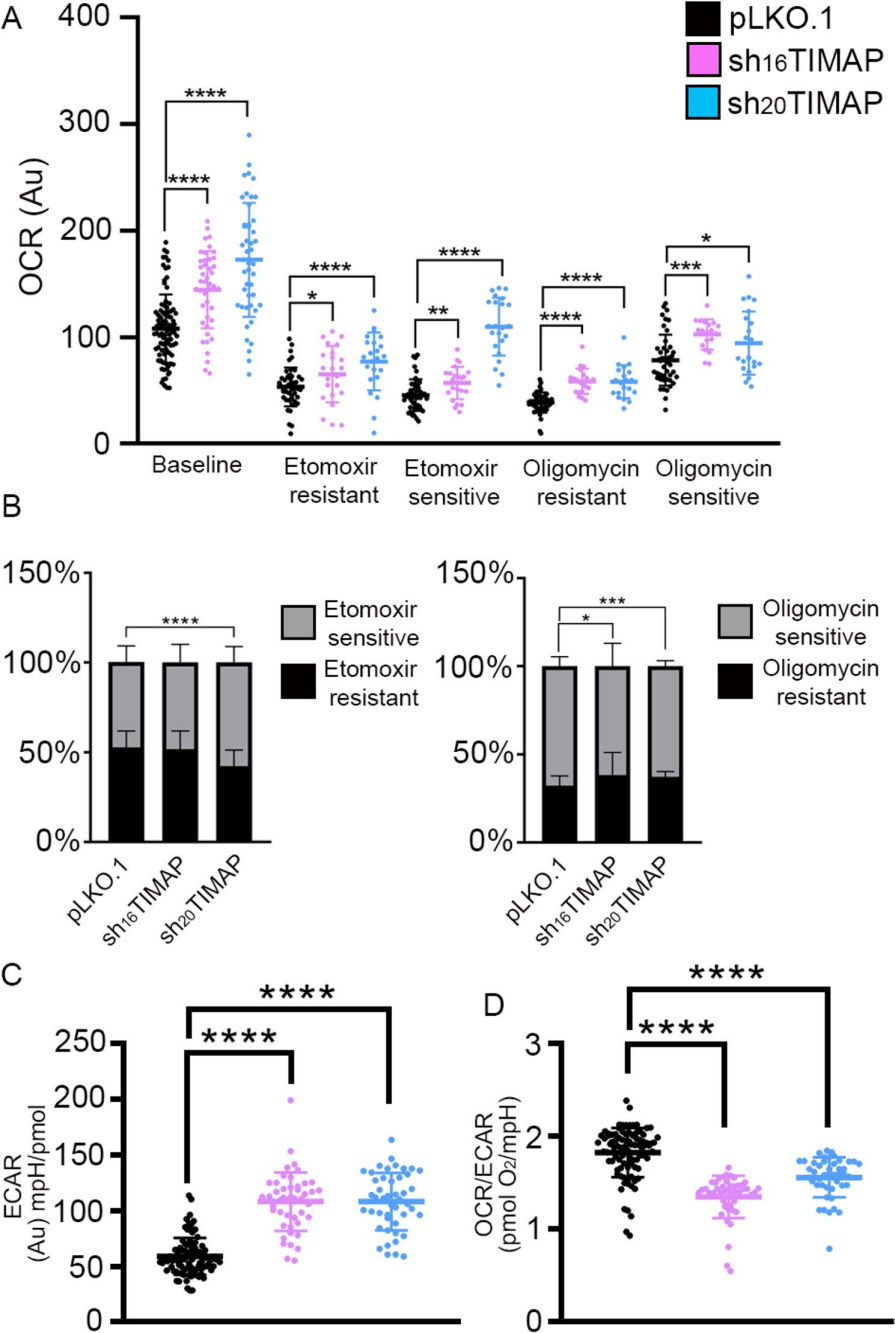
Knockdown of TIMAP renders cells hypermetabolic with a glycolytic dominance.** A** Oxygen consumption rate (OCR) measured under basal conditions and following the addition of etomoxir and oligomycin. Etomoxir-resistant and -sensitive OCR, as well as oligomycin-resistant and -sensitive OCR fractions, are indicated for pLKO.1 control, sh_16_TIMAP and sh_20_TIMAP cells. Statistical analysis was performed using unpaired t-test (*n* = 19–90, ****p < 0.0001, ***p < 0.001, **p < 0.01 and *p < 0.05). **B** Quantification of etomoxir-resistant vs. etomoxir sensitive OCR and oligomycin-resistant vs. oligomycin-sensitive OCR in pLKO.1 and shTIMAP cells. Data are presented as mean ± SD, statistical analysis was carried out using unpaired t-test (*n* = 20–42, ****p < 0.0001, ***p < 0.001, *p < 0.05). **C** Extracellular acidification rate (ECAR) of pLKO.1 and shTIMAP cells. **D** OCR/ECAR ratio comparing pLKO.1 and shTIMAP cells. Group comparisons were conducted with unpaired t-test (*n* = 45–92, ****p < 0.0001)

During EMT cells often undergo metabolic changes to support the increased energy requirements associated with their enhanced migration, invasion and survival [62]. To evaluate how TIMAP depletion affects cellular metabolism, we performed Seahorse extracellular flux analysis. OCR serves as a direct measure of oxidative phosphorylation and reflects mitochondrial function, while ECAR is widely used as an indicator of glycolytic flux [63]. These complementary parameters provide insight into the balance between mitochondrial respiration and glycolysis, allowing us to determine how TIMAP depletion reshapes cellular energy metabolism. All Seahorse measurement were normalized to total protein content per well to account for differences in cell number. At baseline, both shTIMAP cell lines exhibited significantly higher OCR compared to pLKO.1 control cells, suggesting elevated mitochondrial activity under resting conditions (Fig. 11A). Etomoxir blocks fatty acid import to mitochondria splits OCR into etomoxir-sensitive (corresponds to fatty acid oxidation) and sensitive respiration (oxidation substrates other than fatty acids). Upon inhibition of fatty acid oxidation etomoxir-sensitive and etomixir-resistant OCR [64] remained significantly higher in sh_20_TIMAP cells, while sh_16_TIMAP cells showed a more modest, but significant increase. Inhibition of mitochondrial ATP synthase with oligomycin [65] further revealed significant effects. The oligomycin-resistant fraction of OCR, corresponding to uncoupled respiration, was significantly increased in both shTIMAP cell lines compared with controls, and alongside, the oligomycin-sensitive fraction showed a significant increase both in sh_16_ and sh_20_TIMAP cells. Consistent with this, partitioning of OCR into etomoxir- and oligomycin-sensitive versus -resistant fractions showed significant shifts in OCR distribution in shTIMAP cells, particularly upon oligomycin treatment (Fig. 11B), supporting altered coupling and mitochondrial respiratory control. Glycolytic activity, assessed by ECAR, was significantly elevated in both shTIMAP cell lines (Fig. 11C). Importantly, the OCR/ECAR ratio was markedly reduced in shTIMAP cell lines, indicating that TIMAP knockout cells turned hypermetabolic with a glycolytic dominance, i.e. a relative shift toward a glycolytic phenotype (Fig. 11D). These findings suggest that TIMAP knockdown promotes a bioenergetic reprogramming characterized by increased glycolysis and altered mitochondrial respiration, features commonly associated with metabolic plasticity and EMT-related processes of A549 cells [66].

### TIMAP depletion promotes cancer stem cell marker expression

**Fig. 12:**
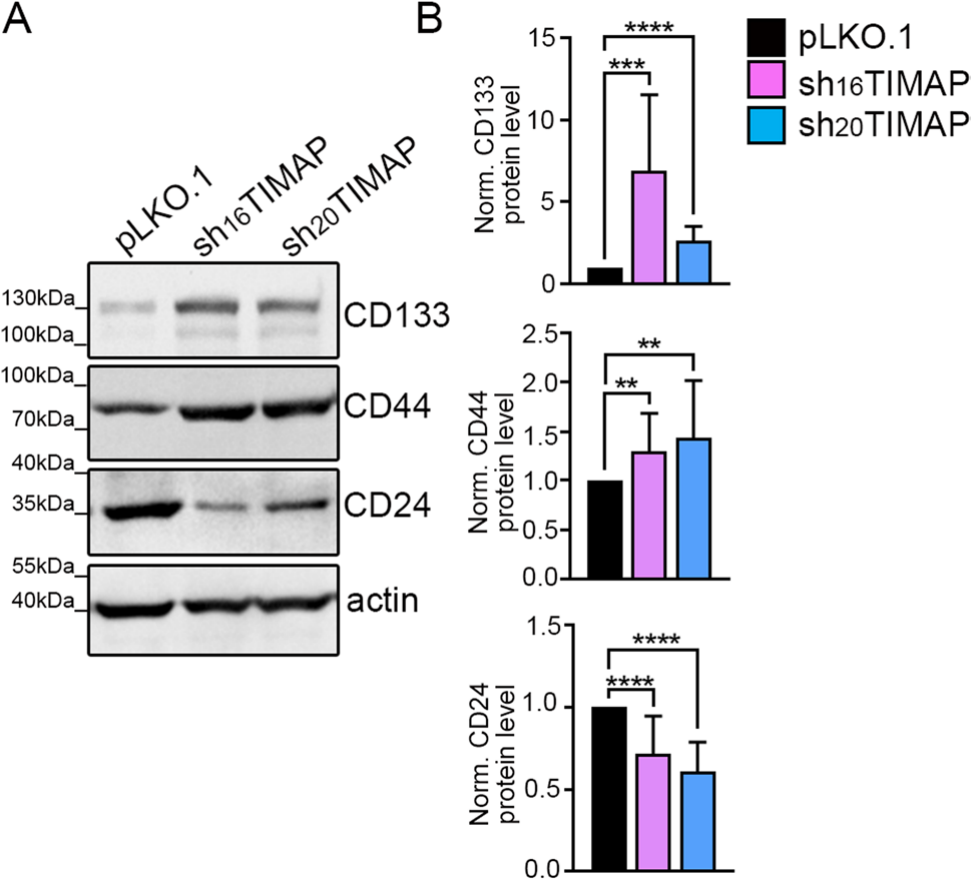
TIMAP depletion alters the protein expression of stem cell markers. **A** Cell lysates derived from pLKO.1, sh_16_TIMAP and sh_20_TIMAP A549 cells were subjected to Western blot analysis with antibodies targeting stem cell markers. **B** Signal intensity was quantified using densitometry and normalized to actin as a loading control. Statistical evaluation was carried out using one-way ANOVA (*n* = 4–16, ****p < 0.0001, ***p < 0.001, **p < 0.01)

Another hallmark of EMT is that cells often acquire cancer stem cell (CSC) properties that increases their tumor-initiating potential, invasiveness and therapy resistance [67]. During EMT, markers such as CD44 and CD133 are typically upregulated, reflecting a mesenchymal, stem-like phenotype, while CD24 expression decreases, consistent with loss of epithelial traits. To investigate whether TIMAP depletion influences these changes, we analysed these typical CSC markers by Western blot (Fig. 12A). As expected, both shTIMAP cell lines displayed a marked downregulation of CD24, while CD44 and CD133 expression was significantly upregulated (Fig. 12B).

### TIMAP expression is reduced in LUAD and correlates with patient outcome

**Fig. 13:**
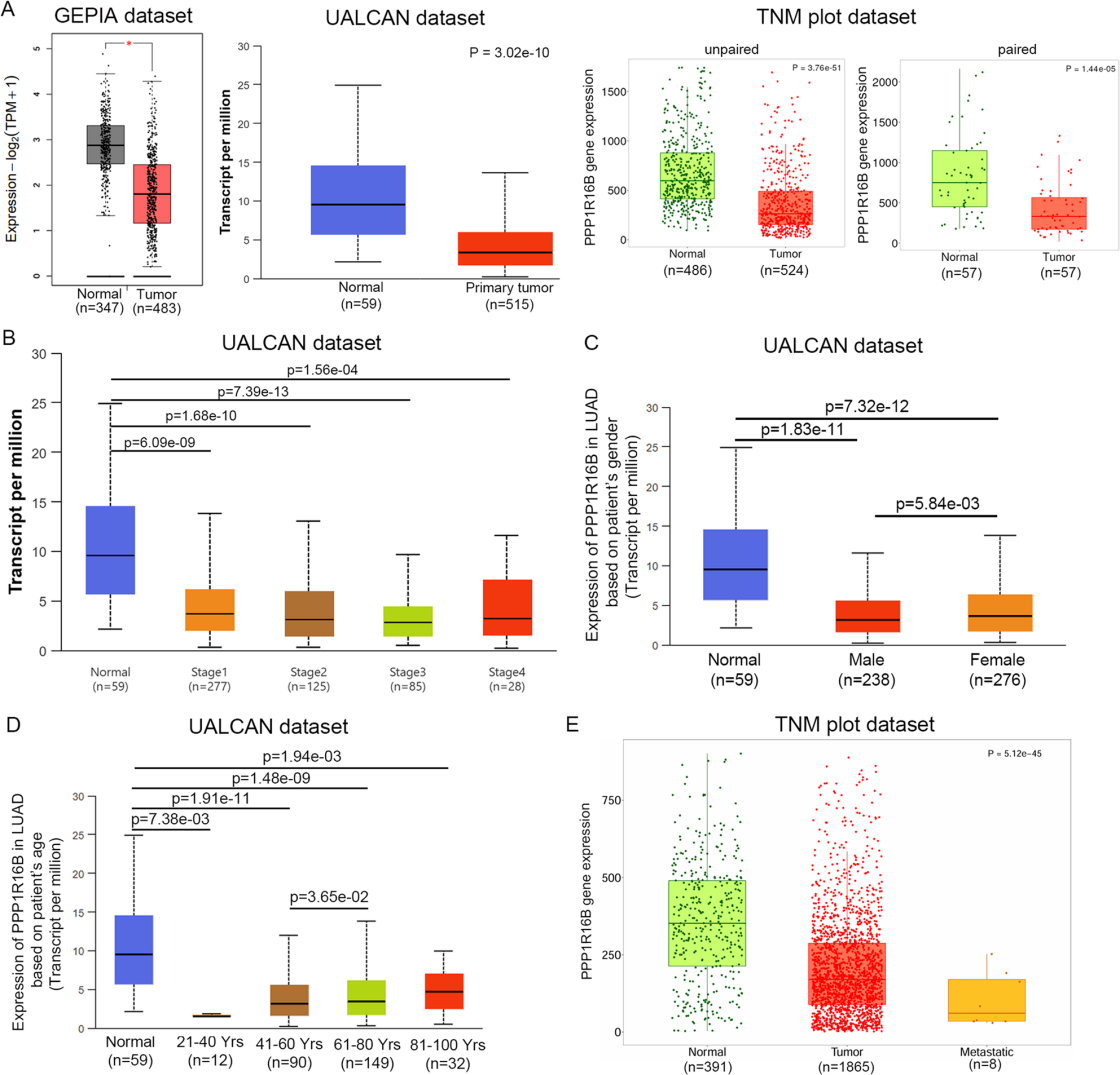
PPP1R16B expression decrease in LUAD. **A** Expression of PPP1R16B was compared between normal and tumor lung tissues using GEPIA, UALCAN and TNMplot databases. **B** Stage-specific expression of PPP1R16B in LUAD was analysed using UALCAN database. **C** Expression of PPP1R16B was compared between normal and tumor male and female lung tissues using UALCAN database. **D** Age-specific expression of PPP1R16B in LUAD was analysed using UALCAN database. **E** Box plot from TNMplot database illustrate expression levels of PPP1R16B in normal, tumor and metastatic samples

To assess the clinical relevance of TIMAP expression (PPP1R16B) in LUAD, we utilized publicly available gene expression databases, including GEPIA, UALCAN and TNMplot. First, we compared PPP1R16B expression between tumor and normal tissues (Fig. 13A). GEPIA analysis revealed a markedly lower transcript level in tumor versus normal controls. Similarly, UALCAN data showed a strong downregulation of PPP1R16B in primary tumor compared to normal tissues (*p* = 3.02e-10). TNMplot analysis confirmed this finding both in unpaired comparisons (*p* = 3.76e-51) and in matched paired tumor-normal samples (*p* = 1.14e-05). Furthermore, PPP1R16B downregulation occurs is early stages of LUAD, as expression was already significantly reduced in stage I tumors (*p* = 6.09e-09 vs. normal) and remained consistently low across advanced stages (Fig. 13B). Stratification by sex showed that PPP1R16B expression was significantly lower in both male (*p* = 1.83e-11) and female (*p* = 7.32e-12) LUAD patients relative to normal controls, with a modest but significant difference observed between male and female tumor samples (*p* = 5.84e-03) (Fig. 13C). Age-stratified analysis further demonstrated significant differences in PPP1R16B expression across age groups (Fig. 13D). Compared with normal tissue, PPP1R16B expression was significantly reduced across all age groups examined. In addition, pairwise comparisons revealed significant difference in expression between the 41–60 and 61–80 age groups (*p* = 3.65e-02). TNMplot analysis further supported this trend, showing robust downregulation in tumor, with expression levels being further reduced in metastatic samples compared to normal tissues (*p* = 5.12e-45) (Fig. 13E).

**Fig. 14:**
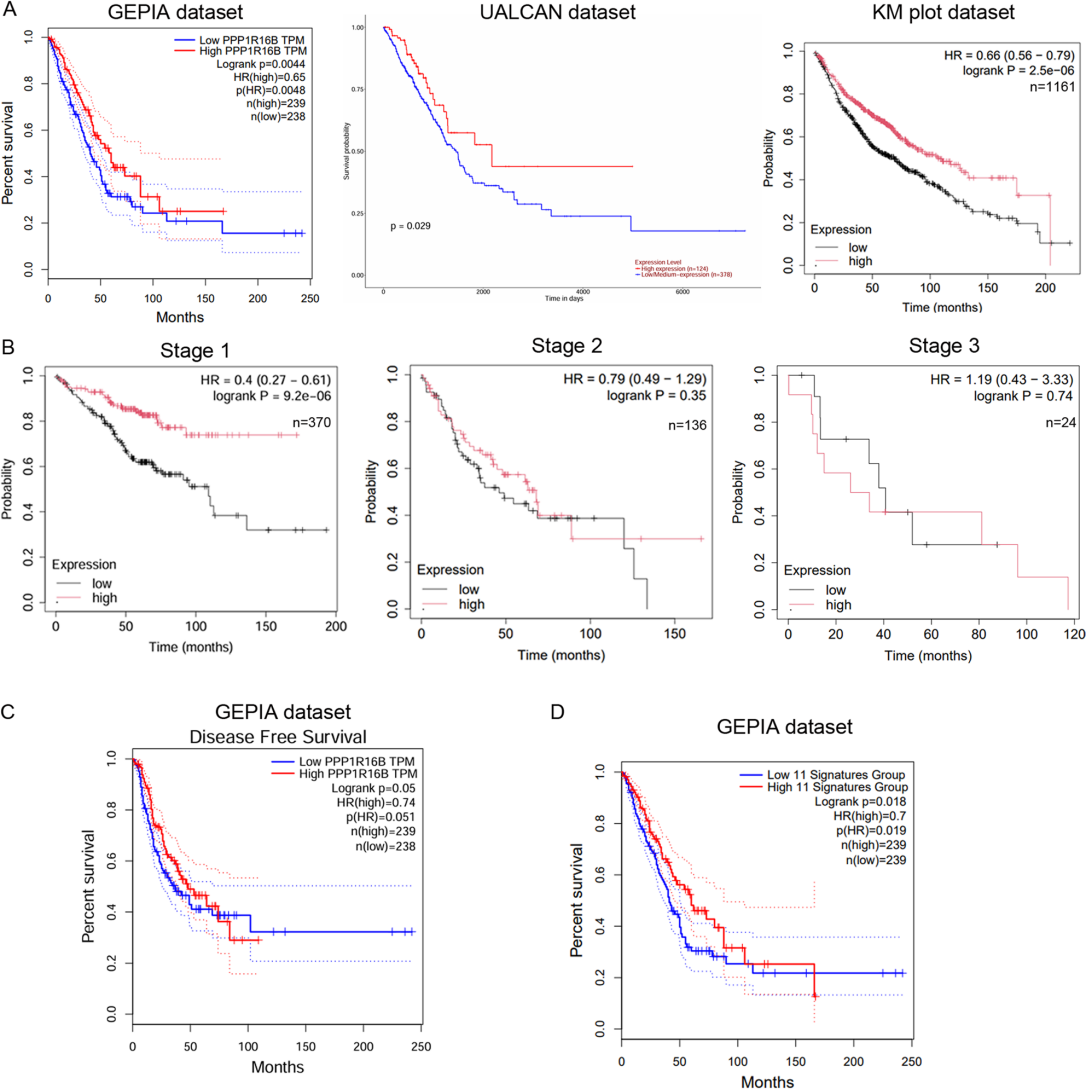
Survival analysis of PPP1R16B in LUAD. **A** Overall survival analysis of PPP1R16B expression in LUAD using GEPIA, UALCAN and KM Plotter datasets. **B** Stage-stratified survival analysis of PPP1R16B expression in LUAD using KM Plotter. **C** Disease-free survival analysis based on PPP1R16B expression using GEPIA **D **Overall survival analysis of a PPP1R16B-related 11 gene signatures identified from downregulated genes in our RNA-seq dataset and validated in UALCAN. Survival analysis was performed in GEPIA using this gene dataset

Kaplan-Meier survival analyses consistently revealed that reduced PPP1R16B expression was associated with significantly worse overall survival (Fig. 14A). In the GEPIA dataset, patients with low TIMAP expression had inferior survival outcomes compared to those with high expression (HR = 0.65, *p* = 0.0048). UALCAN analysis further confirmed a survival benefit in the high-expression group (*p* = 0.029), and KM Plotter analysis corroborated this association (HR = 0.66, 95% CI: 0.56–0.79, *p* = 2.5e-06). Stage-stratified analysis revealed that the prognostic impact of PPP1R16B was most pronounced in stage I patients (HR = 0.40, 95% CI: 0.27–0.61, logrank *p* = 9.2e-06), indicating a ~ 60% reduction in mortality risk in the high-expression group (Fig. 14B). In contrast, no significant survival association was observed in stage II (HR = 0.79, *p* = 0.35) or stage III disease (HR = 1.19, *p* = 0.74).

Disease-free survival (DFS) refers to the length of time after primary treatment during which a patient remains free of any signs or reoccurrence of cancer. In our analysis, DFS using GEPIA demonstrated that patients with high PPP1R16B expression exhibited prolonged DFS compared to those with low expression (HR = 0.71, *p* = 0.051) (Fig. 14C). Although this did not reach conventional statistical significance, there was a clear separation of survival curves, with the high-expression group showing a reduced rate of recurrence over time. The trend suggests that elevated PPP1R16B expression confers a protective effect against disease relapse. In addition, we derived a prognostic 11-gene signature by intersecting PPP1R16B-correlated genes from UALCAN with differentially downregulated genes identified in our RNA-seq dataset (ABCB1, ADAP2, CALHM2, CAMK4, COL14A1, FRMD4B, ICOSLG, IGSF10, KIAA0040, SYK, TLR4). Survival analysis in GEPIA revealed that LUAD patients with high signature expression had significantly better overall survival compared to the low group (*n* = 239, HR = 0.67, log-rank *p* = 0.018) (Fig. 14D). These results collectively demonstrate that PPP1R16B/TIMAP expression is significantly downregulated in LUAD and that its loss occurs early in disease progression. Importantly, reduced TIMAP levels are associated with poor clinical outcomes, particularly in early-stage patients, highlighting its potential utility as a prognostic biomarker and possible therapeutic target in LUAD.

## Discussion

Although, TIMAP has been mainly studied in endothelial cells due to its high expression, emerging evidence supports important regulatory roles in other cell types as well [33, 68–70]. In LUAD, integrative analysis of methylation profiles from identified CpG sites and associated genes with strong prognostic relevance, including TIMAP [71]. In A549 human lung adenocarcinoma cell line, the silencing of TIMAP induced EMT and supported multiple features of tumor progression, such as enhanced cell adhesion and migration, accompanied by morphological changes characteristic of mesenchymal-like cells, increased invasiveness and the formation of unstable spheroids. These alterations are consistent with EMT, wherein epithelial tumor cells lose polarity and intercellular junctions while acquiring motile and invasive properties [7]. TIMAP depletion led to broad perturbations in canonical EMT-associated cascades. Increased phosphorylation of SMAD2/3 indicated activation of TGF-β axis [72], while changes in Wnt signaling were evident from elevated β-catenin phosphorylation at Ser552 and Ser675 [53]. These phosphorylation events promote β-catenin stabilization, nuclear localization, and transcriptional activation in conjunction with TCF/LEF family members [73–75]. Parallel changes were observed in the PI3K/Akt pathway, where increased phosphorylated Akt support the activation of EMT-related signalling cascades in response to TIMAP depletion [53]. Indeed, RNA-sequencing combined with enrichment analysis further validated these observations, revealing robust upregulation of EMT-related gene sets. Epithelial genes such as CDH19 and CLDN3 were significantly downregulated, while mesenchymal and EMT-associated genes (including MMP2, FN1, SNAI1, SNAI2, and ZEB2) were upregulated. Consistent with a mesenchymal shift, we observed changes in the expression of EMT hallmark genes or their protein products, as the downregulation of the epithelial marker E-cadherin [12] and the upregulation of Snail and Slug, which are known to directly repress CDH1 (E-cadherin) expression [76–78]. Snail and Slug are tightly controlled by upstream signalling pathways, including TGF-β, Wnt, and PI3K/Akt, all of which were perturbed following TIMAP depletion [79]. We also observed the characteristic cadherin switching due to TIMAP depletion, with loss of E-cadherin and upregulation of N-cadherin. This shift weakens intercellular adhesion while promoting new ones that favour migration and enhance cell-ECM adhesion [80]. The same trend was observed following TIMAP depletion, with enhanced cell-ECM adhesion and increased migratory capacity, as demonstrated by ECIS measurements. While seemingly contradictory, several reports have shown that claudin-1 overexpression in different tumors may enhance cancer cell invasiveness by promoting EMT-like behaviour, suggesting that claudin-1 upregulation in shTIMAP cells may contribute to EMT through aberrant junctional remodelling rather that maintaining epithelial integrity [81–84]. Further supporting the EMT phenotype, ZO-1 protein levels were reduced, in line with prior findings implicating ZO-1 in migratory signalling [85]. Cytokines are known to promote EMT, inflammation, and extracellular matrix remodelling [86–88] and TIMAP depletion also altered the cytokine secretion profile of cells. The elevated level of uPAR and OPN can contribute to ECM remodelling, while MMP-9 can directly degrade ECM components, enhancing invasion and metastatic potential [89–95]. Elevated levels of CXCL1, CXCL5, CXCL8, MCP-1, PDGF-AA and MIF was also shown and are associated with EMT, migration and tumor progression in LUAD and other neoplasia [59–61]. Secreted Dkk-1 was also elevated following TIMAP loss, consistent with its reported role as a modulator of Wnt signaling and its association with tumor progression and aggressiveness in non-small cell lung cancer [96, 97]. Conversely, the downregulation of IGFBP-2 and LCN2 may reflect reduced differentiation signals and a shift toward a more aggressive state [98, 99]. Although IGFBP2 has been implicated as a pro-EMT factor in various cancers [100], its downregulation removes its inhibitory effect on IGFR1 and can activate Akt-mTORC2 signalling, leading to hyper glycolytic phenotype [101, 102]. Beyond the classical role in iron metabolism, LCN-2 has been associated with EMT [103]. LCN-2 can suppress EMT by maintaining epithelial characteristics and suppress invasive behaviour [99, 104]. IGFBP-2 is a modulator of IGF signalling throughout the regulation of differentiation and survival and been implicated in maintaining epithelial cell characteristic and restraining tumor aggressiveness [105, 106]. In cancer cells, IGFBP-2 normally restrains IGFR1 signaling and its downregulation relieves this inhibition, resulting in an enhanced Akt-mTORC2 pathway [107]. This hyperactivation can lead to an altered metabolic state of cells. Interestingly TIMAP induced a profound metabolic reprogramming, shifting cellular energy utilization from oxidative phosphorylation toward glycolysis. This bioenergetic switch is also a hallmark of EMT and cancer progression [108]. The observed reduction in the OCR/ECAR ratio in shTIMAP cells is consistent with enhanced glycolytic dependency, that is linked to increased migratory potential and survival [109]. In parallel, TIMAP depletion promoted the acquisition of CSC-like features, as evidenced by upregulation of CD44 and CD133 together with loss of CD24 expression. These shifts in marker expression are well recognized signatures of stem-like, mesenchymal states that enhance tumor-initiation capacity and invasiveness or affect therapeutic resistance [67, 110]. Although EMT promotes enhanced migration through cytoskeletal reorganization and loss of epithelial junctions, it simultaneously slows proliferation via EMT-associated TFs that modulate the cell cycle and destabilize p53 [111]. This combination of slower division and increased motility is tightly linked to the acquisition of CSC-like properties, supporting both dissemination and metastatic potential [112]. Collectively, these results suggest that TIMAP loss contributes to a tumor-promoting microenvironment by modulating EMT, cytokine secretion, and cell plasticity.

Analysis of clinical datasets revealed that TIMAP levels are significantly reduced in tumor tissues compared to normal lung. TIMAP downregulation takes place in the early stages of the disease. Furthermore, high tumoral TIMAP expression is associated with improved overall and DFS in lung adenocarcinoma patients. Such stage-specific associations highlight TIMAP as a candidate early biomarker with both diagnostic and prognostic relevance. Together with our experimental findings in A549 cells, these results support a tumor-suppressive role for TIMAP in lung cancer.

## Conclusions

In conclusion, our findings establish TIMAP as a critical regulator of EMT and cellular plasticity in LUAD. By influencing both transcriptional and post-transcriptional networks, TIMAP helps maintain epithelial identity. TIMAP loss promotes metabolic reprogramming and acquisition of stem-like traits, contributing to disease progression and poor clinical outcomes, and highlights TIMAP as a potential target for strategies.

## Supplementary Information

Supplementary Material 1: Suppl. Figure 1. Depletion of TIMAP alters EMT marker expression in the SK-LU-1 cell line (A) SK-LU-1 cell lysates from pLKO.1, sh16TIMAP and sh20TIMAP cells were analysed by Western blot using antibodies against TIMAP and the EMT markers Snail, Slug, E-cadherin and N-cadherin. Actin was used as a loading control (B) Band intensities were quantified by densitometry and normalized to actin. Statistical analysis was performed using one-way ANOVA (n=3-4, ****p<0.0001, ***p<0.001, **p<0.01, *p<0.05).

## Data Availability

The raw data underlying the figures are available on Figshare (DOI: 10.6084/m9.figshare.31177102).The datasets used and/or analysed during the current study are available from the corresponding author on reasonable request.
