# Supplementary material for: Loss of protein phosphatase 1 regulator TIMAP protein triggers EMT in A549 cells
Source: Cell Commun Signal. 2026 Apr 13;24:321. doi: 10.1186/s12964-026-02873-5 (PMC13217683; doi:10.1186/s12964-026-02873-5)

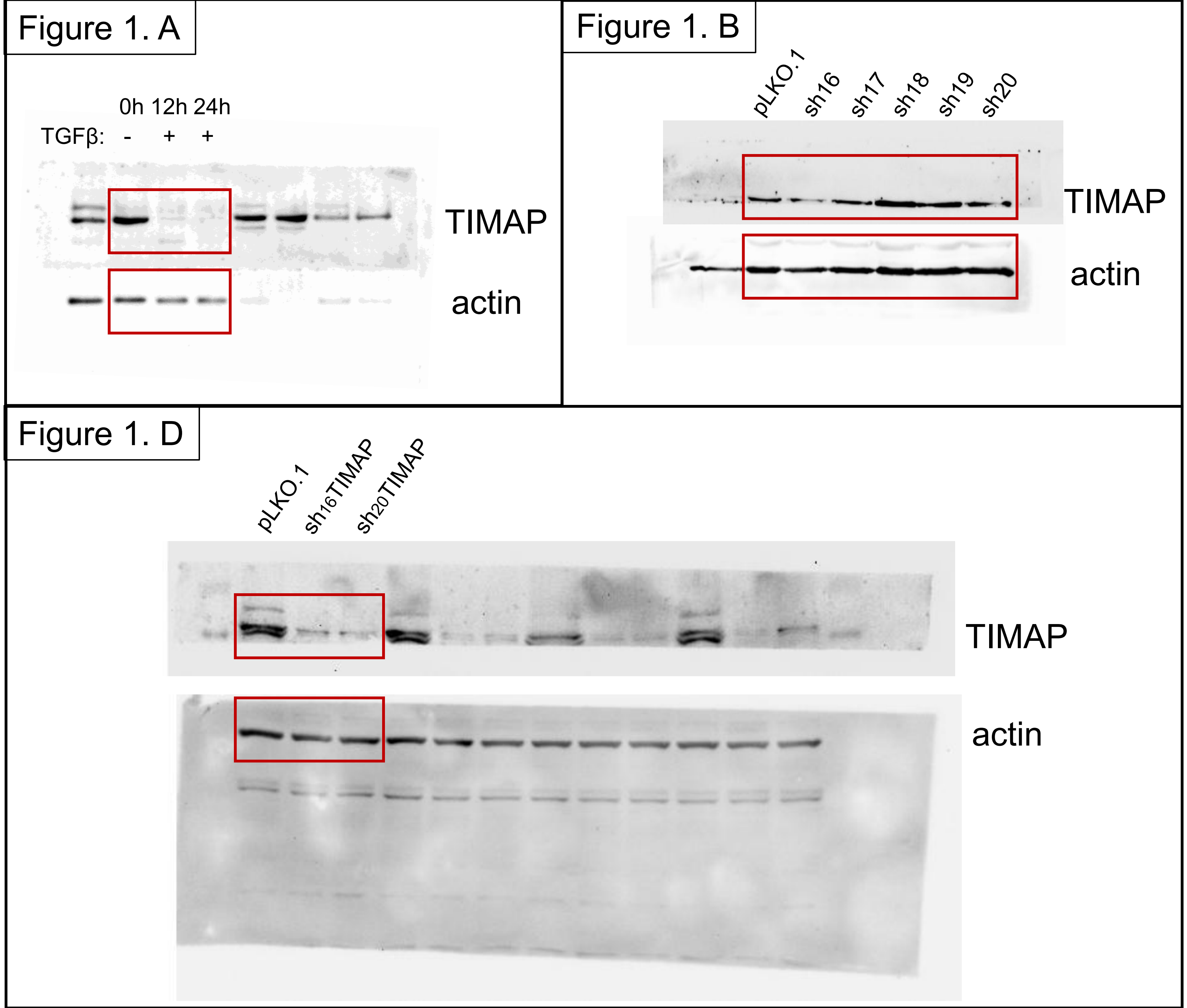

Figure 1. A
0h 12h 24h
TGFβ: - + +
TIMAP
actin
Figure 1. B
pLKO.1
sh16
sh17
sh18
sh19
sh20
TIMAP
actin
Figure 1. D
pLKO.1
sh16TIMAP
sh20TIMAP
TIMAP
actin

Figure 4. B

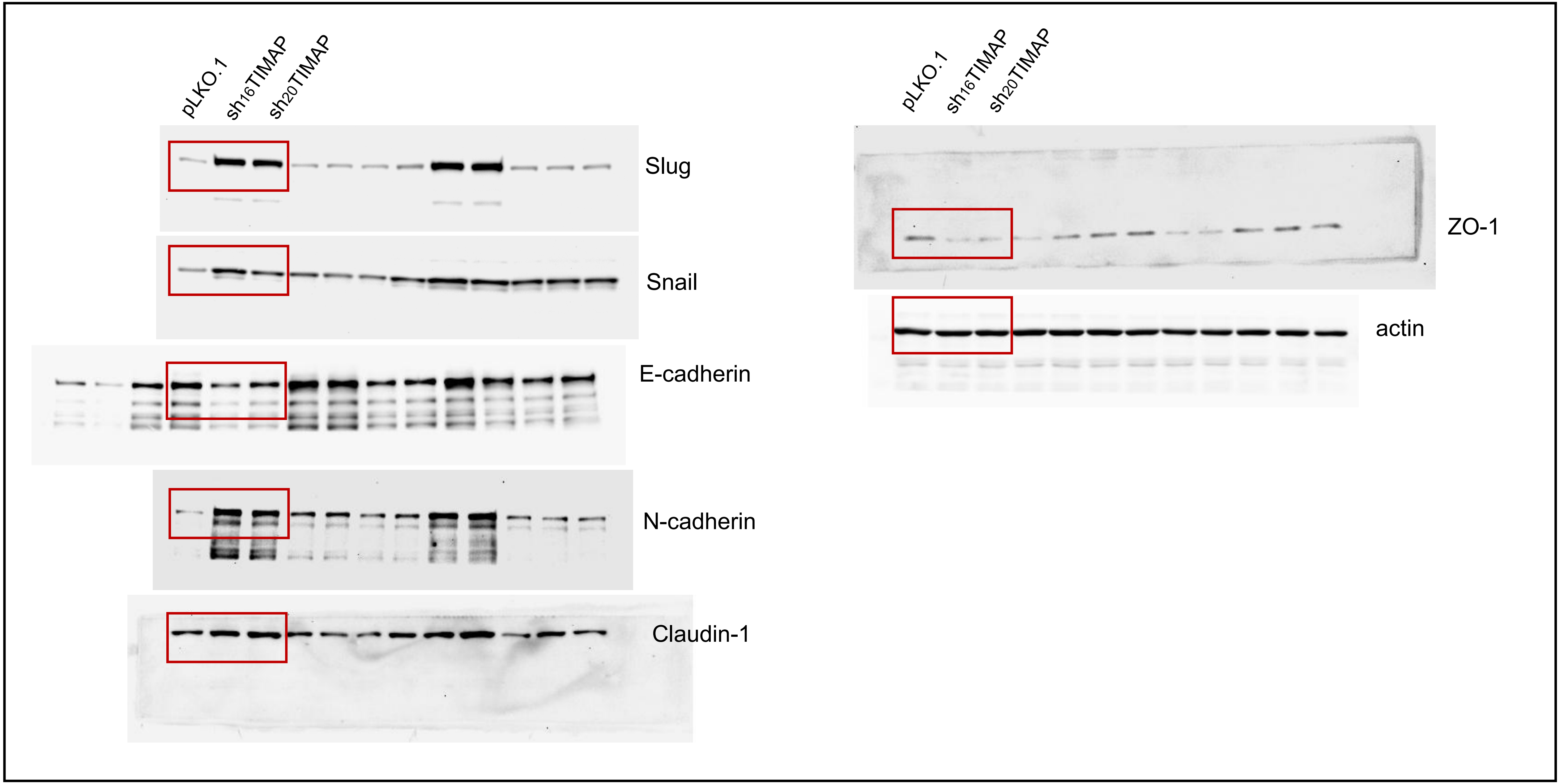
pLKO.1
sh16TIMAP
sh20TIMAP
Slug
Snail
E-cadherin
N-cadherin
Claudin-1
pLKO.1
sh16TIMAP
sh20TIMAP
ZO-1
actin

Figure 5. A

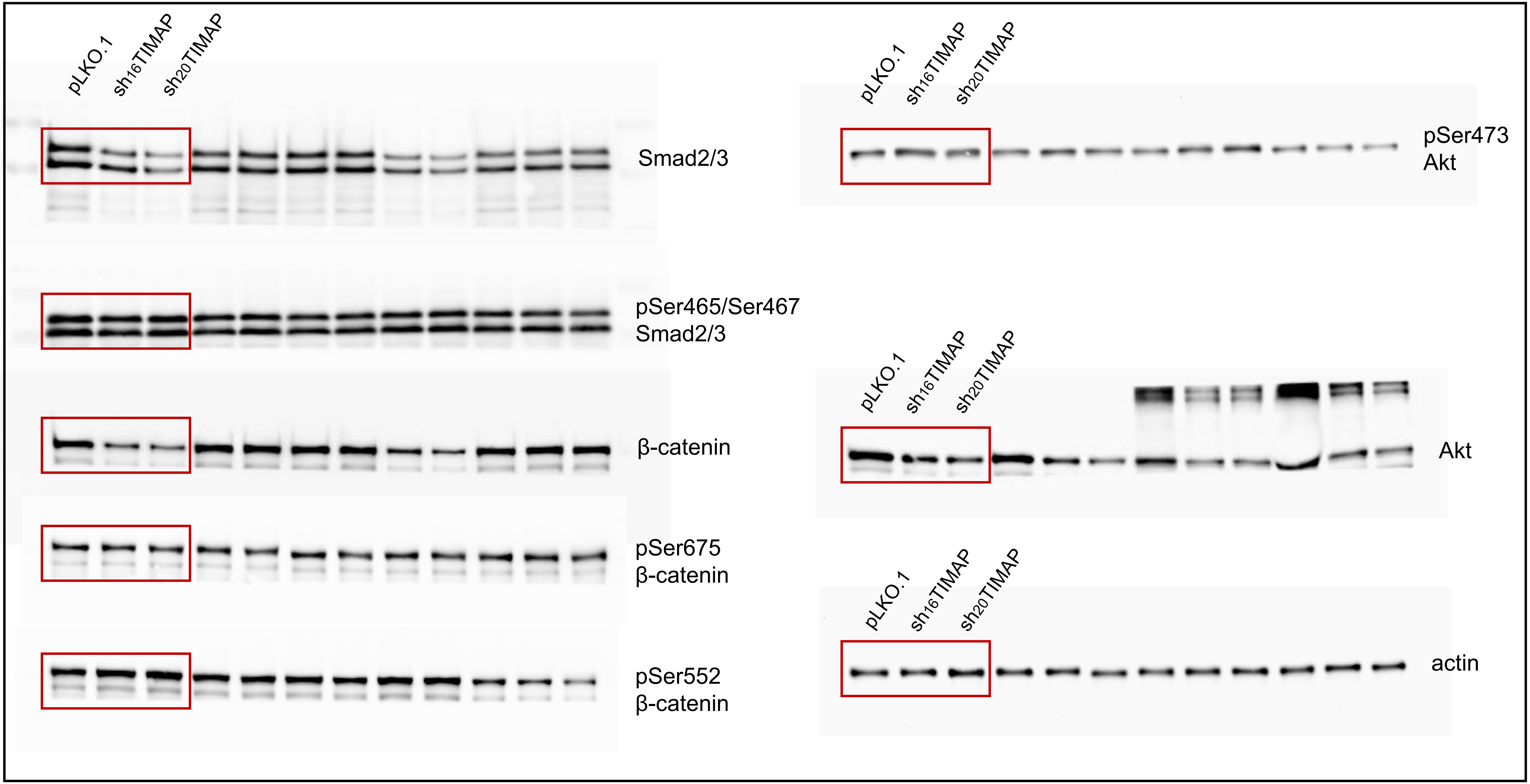
pLKO.1
sh16TIMAP
sh20TIMAP
Smad2/3
pSer465/Ser467
Smad2/3
β-catenin
pSer675
β-catenin
pSer552
β-catenin
pSer473
Akt
Akt
actin

Figure 6. C

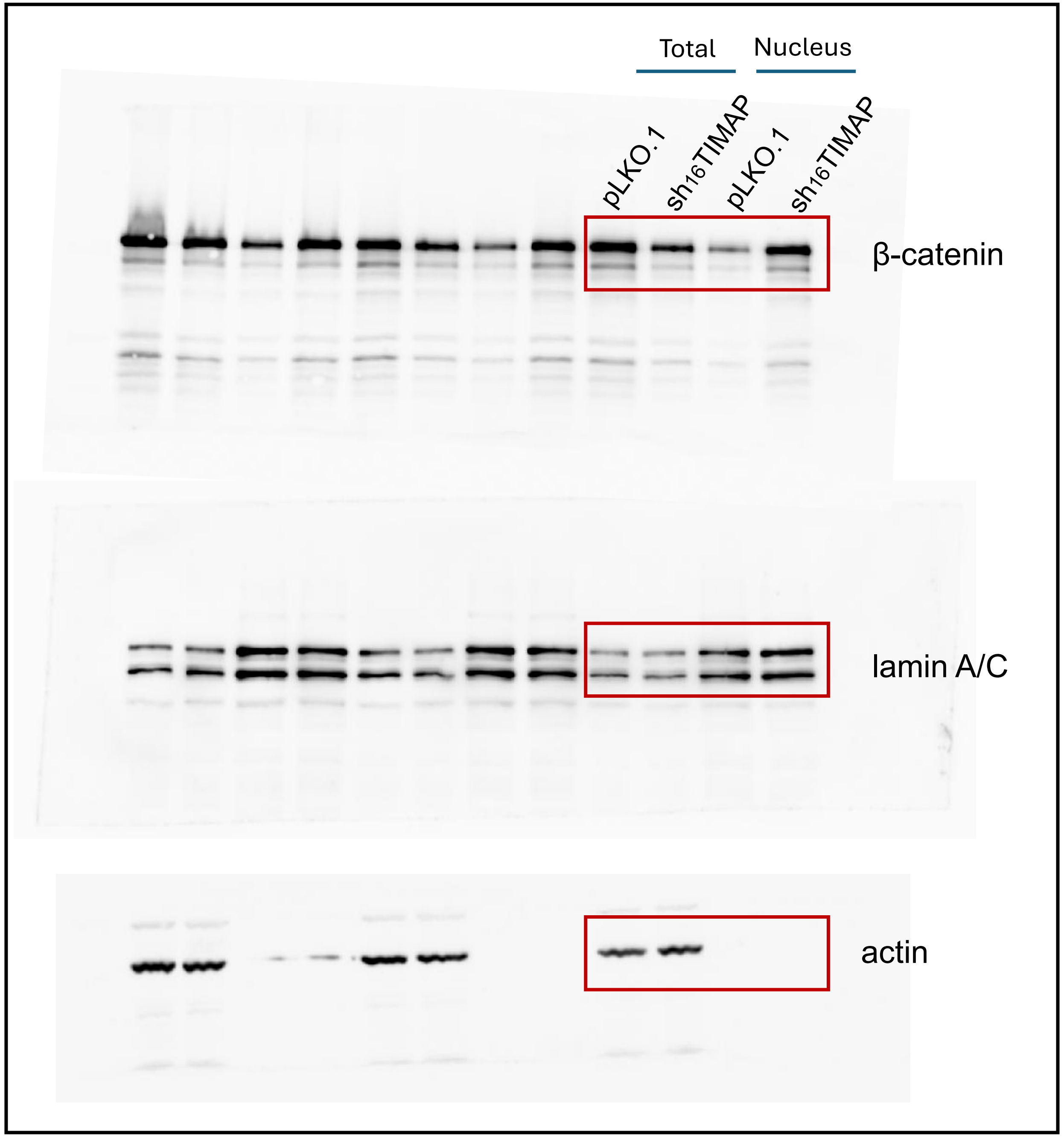
Total
Nucleus
pLKO.1
sh16TIMAP
pLKO.1
sh16TIMAP
β-catenin
lamin A/C
actin

Figure 7. A

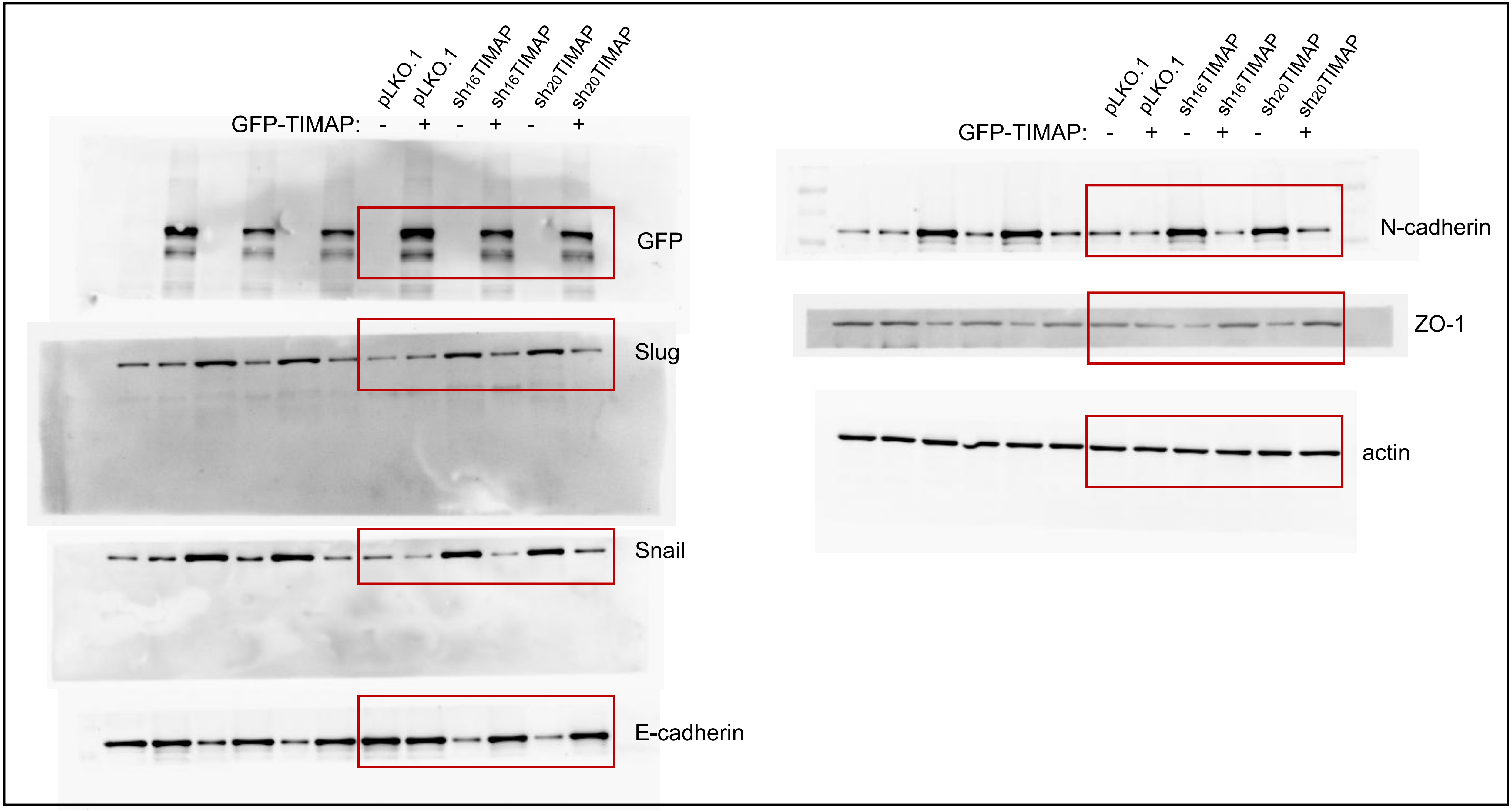

pLKO.1
pLKO.1
sh16TIMAP
sh16TIMAP
sh20TIMAP
sh20TIMAP
GFP-TIMAP: - + - + - +
GFP
Slug
Snail
E-cadherin
pLKO.1
pLKO.1
sh16TIMAP
sh16TIMAP
sh20TIMAP
sh20TIMAP
GFP-TIMAP: - + - + - +
N-cadherin
ZO-1
actin

Figure 8. A

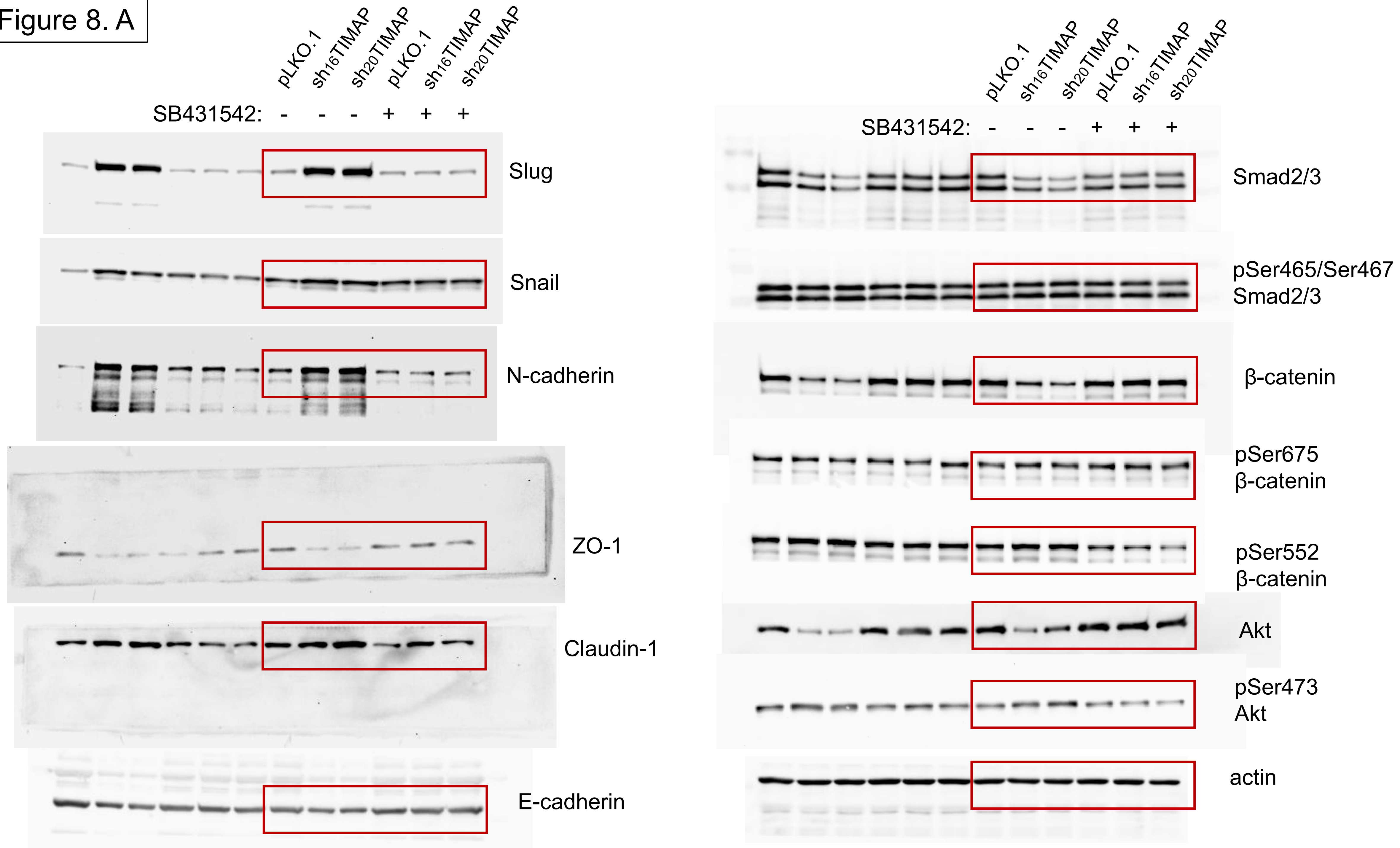
pLKO.1
sh16TIMAP
sh20TIMAP
pLKO.1
sh16TIMAP
sh20TIMAP
SB431542: - - - + + +
Slug
Snail
N-cadherin
ZO-1
Claudin-1
E-cadherin
pLKO.1
sh16TIMAP
sh20TIMAP
pLKO.1
sh16TIMAP
sh20TIMAP
SB431542: - - - + + +
Smad2/3
pSer465/Ser467
Smad2/3
β-catenin
pSer675
β-catenin
pSer552
β-catenin
Akt
pSer473
Akt
actin

Figure 10. A

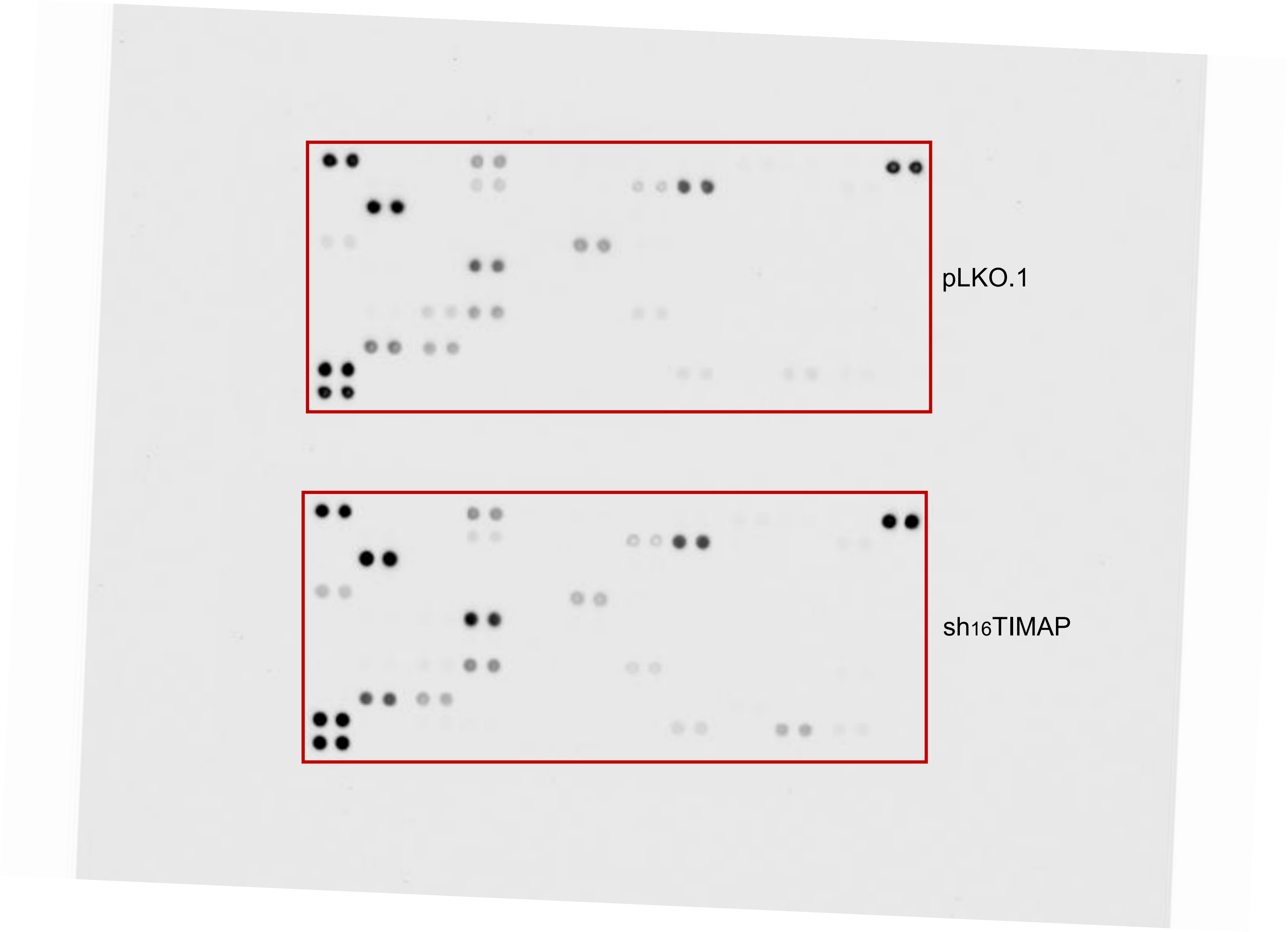

Figure 12. A

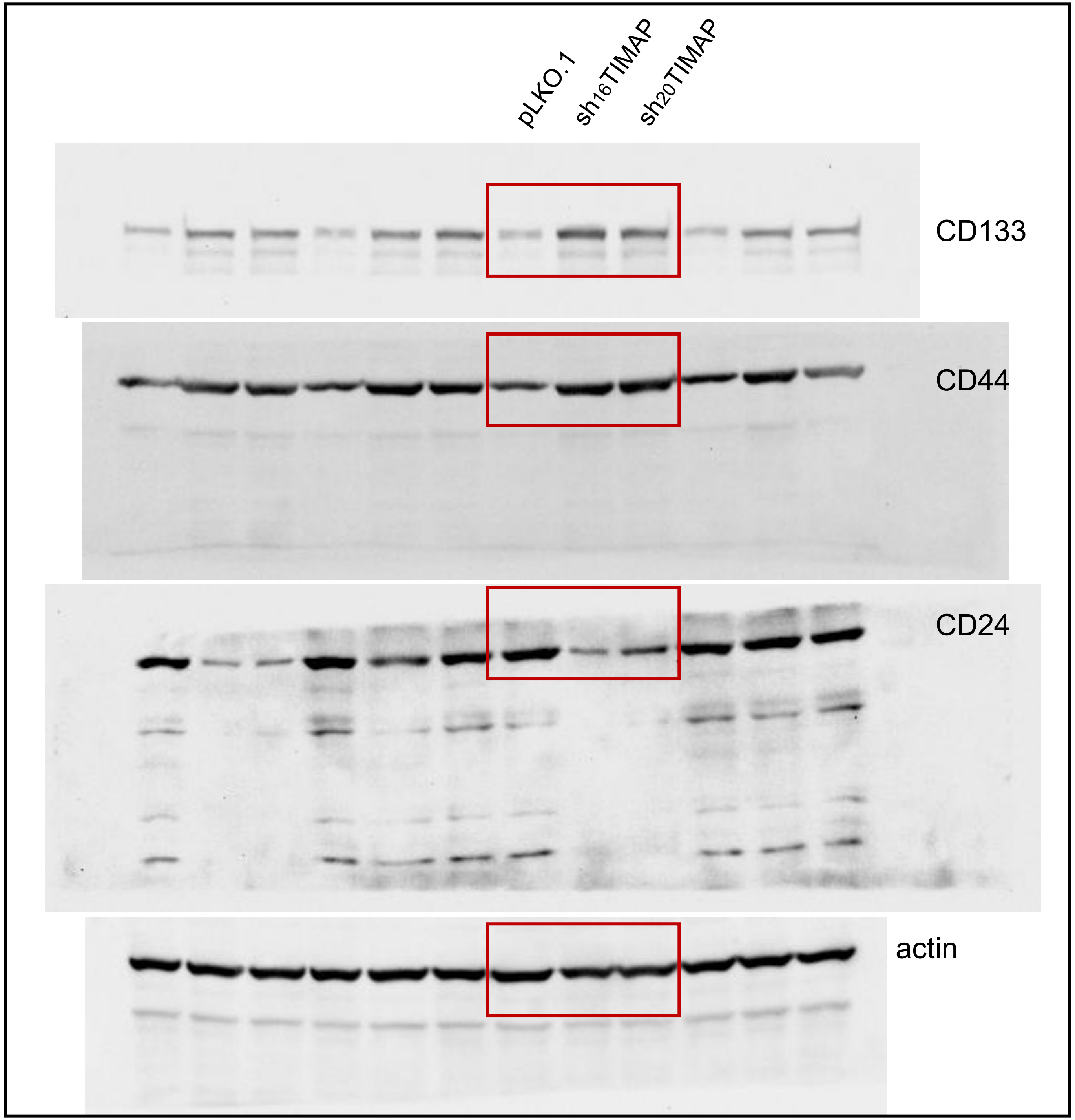

Suppl. Figure 1.

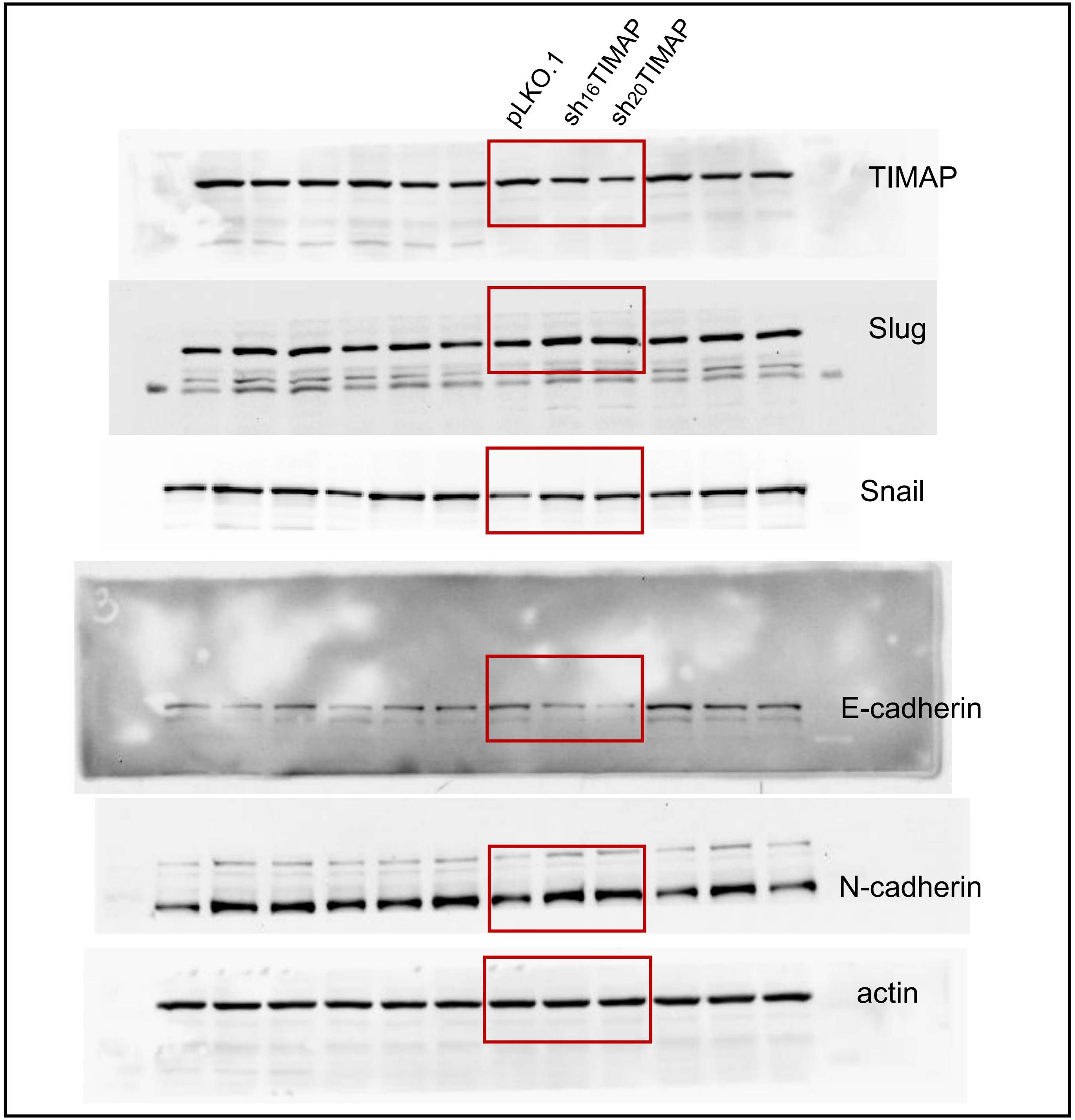

Supplement: Supplementary file 1 — Supplementary Material 1: Suppl. Figure 1. Depletion of TIMAP alters EMT marker expression in the SK-LU-1 cell line (A) SK-LU-1 cell lysates from pLKO.1, sh16TIMAP and sh20TIMAP cells were analysed by Western blot using antibodies against TIMAP and the EMT markers Snail, Slug, E-cadherin and N-cadherin. Actin was used as a loading control (B) Band intensities were quantified by densitometry and normalized to actin. Statistical analysis was performed using one-way ANOVA (n=3-4, ****p<0.0001, ***p<0.001, **p<0.01, *p<0.05). [file 12964_2026_2873_MOESM1_ESM.zip › original uncropped images.pdf]
