# Supplementary material for: Loss of protein phosphatase 1 regulator TIMAP protein triggers EMT in A549 cells
Source: Cell Commun Signal. 2026 Apr 13;24:321. doi: 10.1186/s12964-026-02873-5 (PMC13217683; doi:10.1186/s12964-026-02873-5)
Supplement: Supplementary file 1 — Supplementary Material 1: Suppl. Figure 1. Depletion of TIMAP alters EMT marker expression in the SK-LU-1 cell line (A) SK-LU-1 cell lysates from pLKO.1, sh16TIMAP and sh20TIMAP cells were analysed by Western blot using antibodies against TIMAP and the EMT markers Snail, Slug, E-cadherin and N-cadherin. Actin was used as a loading control (B) Band intensities were quantified by densitometry and normalized to actin. Statistical analysis was performed using one-way ANOVA (n=3-4, ****p<0.0001, ***p<0.001, **p<0.01, *p<0.05). [file 12964_2026_2873_MOESM1_ESM.zip › FigS1.docx]

**
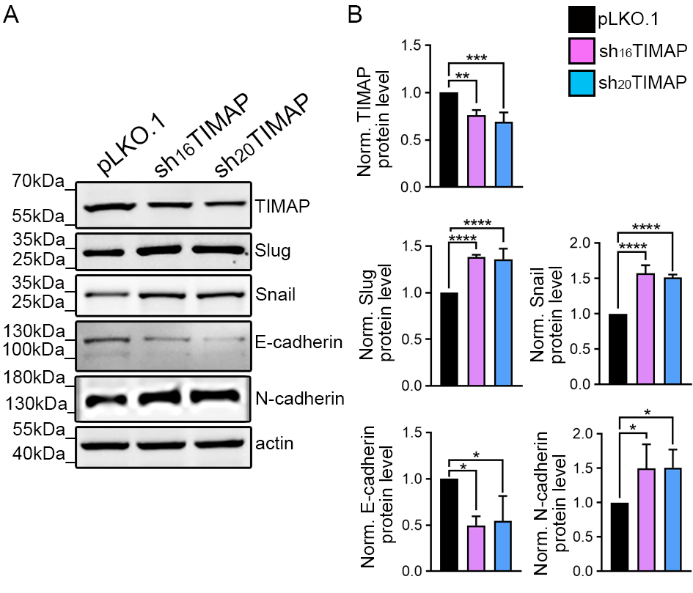
**

**Suppl. Figure 1. Depletion of TIMAP alters EMT marker expression in the SK-LU-1 cell line (A)** SK-LU-1 cell lysates from pLKO.1, sh_16_TIMAP and sh_20_TIMAP cells were analysed by Western blot using antibodies against TIMAP and the EMT markers Snail, Slug, E-cadherin and N-cadherin. Actin was used as a loading control **(B)** Band intensities were quantified by densitometry and normalized to actin. Statistical analysis was performed using one-way ANOVA (n=3-4, *****p<0.0001, ***p<0.001, **p<0.01, *p<0.05*).
